# Platform-independent gene expression signature differentiates sessile serrated adenomas/polyps and hyperplastic polyps of the colon

**DOI:** 10.1186/s12920-017-0317-7

**Published:** 2017-12-28

**Authors:** Yasir Rahmatallah, Magomed Khaidakov, Keith K. Lai, Hannah E. Goyne, Laura W. Lamps, Curt H. Hagedorn, Galina Glazko

**Affiliations:** 1 Department of Biomedical Informatics, University of Arkansas for Medical Sciences, Little Rock, AR 72205 USA; 2 The Central Arkansas Veterans Healthcare System, Little Rock, AR 72205 USA; 3 Department of Medicine, Division of Gastroenterology and Hepatology, University of Arkansas for Medical Sciences, Little Rock, AR 72205 USA; 4 Department of Pathology, University of Arkansas for Medical Sciences, Little Rock, AR 72205 USA; 5 Department of Anatomic Pathology, Cleveland Clinic, Cleveland, OH 44195 USA

**Keywords:** Sessile serrated adenoma/polys, Hyperplastic polyps, Molecular signature, RNA-seq, Microarrays, Formalin-fixed paraffin-embedded, Shrunken centroid classifier, Summary metric, Feature selection, Cantelli’s inequality

## Abstract

**Background:**

Sessile serrated adenomas/polyps are distinguished from hyperplastic colonic polyps subjectively by their endoscopic appearance and histological morphology. However, hyperplastic and sessile serrated polyps can have overlapping morphological features resulting in sessile serrated polyps diagnosed as hyperplastic. While sessile serrated polyps can progress into colon cancer, hyperplastic polyps have virtually no risk for colon cancer. Objective measures, differentiating these types of polyps would improve cancer prevention and treatment outcome.

**Methods:**

RNA-seq training data set and Affimetrix, Illumina testing data sets were obtained from Gene Expression Omnibus (GEO). RNA-seq single-end reads were filtered with FastX toolkit. Read mapping to the human genome, gene abundance estimation, and differential expression analysis were performed with Tophat-Cufflinks pipeline. Background correction, normalization, and probe summarization steps for Affimetrix arrays were performed using the robust multi-array method (RMA). For Illumina arrays, log_2_-scale expression data was obtained from GEO. Pathway analysis was implemented using Bioconductor package GSAR. To build a platform-independent molecular classifier that accurately differentiates sessile serrated and hyperplastic polyps we developed a new feature selection step. We also developed a simple procedure to classify new samples as either sessile serrated or hyperplastic with a class probability assigned to the decision, estimated using Cantelli’s inequality.

**Results:**

The classifier trained on RNA-seq data and tested on two independent microarray data sets resulted in zero and three errors. The classifier was further tested using quantitative real-time PCR expression levels of 45 blinded independent formalin-fixed paraffin-embedded specimens and was highly accurate. Pathway analyses have shown that sessile serrated polyps are distinguished from hyperplastic polyps and normal controls by: up-regulation of pathways implicated in proliferation, inflammation, cell-cell adhesion and down-regulation of serine threonine kinase signaling pathway; differential co-expression of pathways regulating cell division, protein trafficking and kinase activities.

**Conclusions:**

Most of the differentially expressed pathways are known as hallmarks of cancer and likely to explain why sessile serrated polyps are more prone to neoplastic transformation than hyperplastic. The new molecular classifier includes 13 genes and may facilitate objective differentiation between two polyps.

**Electronic supplementary material:**

The online version of this article (10.1186/s12920-017-0317-7) contains supplementary material, which is available to authorized users.

## Background

Screening programs for colorectal cancer (CRC) have resulted in a significant reduction of related deaths [1–4]. Screening programs include the appropriate use of colonoscopy and removal of premalignant polyps. However, colonic polyps differ in their risk for progression to cancer and recommendations for removal and follow-up vary depending on their type [5]. The most common types include the conventional adenomas and serrated polyps, and until approximately 1996 hyperplastic polyps were considered the primary type of serrated polyp [5, 6]. The term sessile serrated polyp was introduced to define serrated lesions prone to progression to cancer, without cytological dysplasia and have been reported in 5–8% of average-risk patients undergoing screening colonoscopy [6–11]. Serrated polyps are divided into three main categories: typical hyperplastic polyps (HPs), SSA/Ps and traditional serrated adenomas (relatively rare) [5]. However, SSA/Ps and HPs share histological similarities. Both types of polyps have the principal feature of a serrated crypt architecture, but SSA/Ps have a histological morphology characterized by dilated, horizontal, and boot-shaped crypts. In general, SSA/Ps are more commonly located in the right colon and are generally larger, flat and hypermucinous [12–15]. However, given the histologic overlap between the two polyp types, biopsy specimens are frequently equivocal in cases lacking the diagnostic hallmarks of SSA/Ps. In addition, several studies have pointed out significant observer-to-observer variability [16, 17]. Because SSA/Ps have the potential to progress into colon cancer [5, 14], reliable biomarkers that aid their diagnosis are needed. It is estimated that SSA/Ps account for 15–30% of colon cancers by progression through the serrated neoplasia pathway [11, 15, 18]. However, this pathway remains relatively uncharacterized as compared to the adenoma to carcinoma pathway. Genetic and epigenetic mechanisms operating in the serrated pathway can include BRAF mutations, KRAS mutations, CpG island methylator high (CIMP-H) and microsatellite instability high (MSI-H) phenotypes which often predict a poor clinical outcome [11, 18, 19]. However, the serrated neoplasia pathway remains to be defined by a characteristic set of genetic and epigenetic lesions.

Since the advent of high-throughput gene expression technologies (microarrays, RNA sequencing) molecular signatures that accurately diagnose or predict disease outcome based on expression of sets of genes have been developed [20, 21]. In many cases gene expression signatures can be associated with biological mechanisms, subtypes of cancer that look histologically similar, tumor stages, as well as the ability to metastasize, relapse or respond to specific therapies [22–27]. Expression-based classifiers were also developed to identify patients with a poor prognosis for stage II colon cancers [28–30]. Recently, a subgroup of colon cancers with a poor prognosis was identified and this subgroup has several up-regulated pathways in common with SSA/Ps [19]. However, there is no molecular classifier differentiating SSA/Ps and HPs.

Several recent studies used transcriptome analyses to gain insights into the biology of SSA/Ps. For example, in a gene array study SSA/Ps were compared to tubular adenomas (TAs) and control samples [31]. Among 67 differentially expressed (DE) genes the two most up-regulated genes (cathepsin E and trefoil factor 1) were verified by quantitative real time reverse transcription PCR (qRT-PCR) and immunohistochemistry analyses showing that these genes were overexpressed in SSA/Ps [31]. In another gene array study 162 DE genes were identified in SSA/Ps as compared to HPs [32]. Validation by qRT-PCR and immunohistochemistry identified annexin A10 as a potential diagnostic marker of SSA/Ps. Another study used RNA sequencing (RNA-seq) to analyze the SSA/P transcriptomes and identified 1294 genes that were differentially expressed in SSA/Ps as compared to HPs. This analysis provided evidence that molecular pathways involved in colonic mucosal integrity and cell adhesion were overrepresented in SSA/Ps [33].

The goals of our study were two-fold. We aimed to gain insight into the biological differences between SSA/Ps and HPs and develop a gene expression-based classifier that reliably differentiated HPs and SSA/Ps. We analyzed data from HPs and SSA/Ps, with unequivocal diagnoses, matched with control samples. Notably, the right and left colon have different embryological origins and more than 1000 genes are differentially expressed between the adult right and left colon [34]. SSA/Ps occur predominantly in the right colon and HPs occur predominantly in the left colon. Consequently, some genes that are DE between SSA/Ps and HPs are likely to be due to their frequently different anatomical location (SSA/Ps right and HPs left). To find genes and pathways that are DE specifically between SSA/Ps and HPs, we hypothesized that it was first necessary to exclude genes that are DE between the right and left colon. In addition to SSA/P and HP specimens, control samples obtained from the right colon (CR) and left colon (CL) were also included in the study. The analysis of differentially expressed genes and pathways revealed several differentially expressed and differentially co-expressed pathways between SSA/Ps and HPs, CR specimens. The pathways found here are generally considered hallmarks of cancer: they were associated with the ability to escape apoptotic signals, the inflammatory state of premalignant lesions and uncontrolled proliferation [35].

Our second aim was to develop a gene expression-based classifier that reliably differentiates HPs and SSA/Ps and is platform-independent (works for RNA-seq as well as microarrays). Independent microarray data sets were collected: an Illumina gene array data set [32] (six HPs and six SSA/Ps) and subsets of samples from two Affymetrix data sets (eleven HPs from GSE10714 [36] and six SSA/Ps from GSE45270 [19]). Typically, the most ambiguous step in classifier development is the step of feature selection because of the ‘large *p* small *n*’ problem of omics data [37]. Omics data have at most only hundreds of samples (*n*) and thousands of features (*p*), and using all features will lead to model over-fitting and poor generalizability. Feature selection techniques differ in the way they combine feature selection with the construction of the classification model and usually are classified into three categories: filter, wrapper, and embedded algorithms [37]. Filter algorithms preselect features before using classifier based, for example, on the results of significance testing. Wrapper algorithms combine the search of optimal features with the model selection and evaluate features by training and testing classification model. For example, the Shrunken Centroid Classifier (SCC) first finds a centroid for each class and selects features to shrink the gene centroid toward the overall class centroid [38]. Here we presented a new way to combine filter and wrapper algorithms that fitted best to our goal of building a platform independent classifier. First, we reduced the feature space by selecting only those features (genes) that were concordantly expressed over all three platforms. Second, we applied SCC (using all genes left after filtering) on RNA-seq data for further reducing the feature space and selecting features with optimal classification performance. The classifier, developed based on RNA-seq data identified SSA/P and HP subtypes in independent microarray data sets with low classification errors. The molecular signature that correctly classifies SSA/Ps and HPs consists of thirteen genes and is a first platform-independent signature that could be applied as a diagnostic tool for distinguishing SSA/Ps from HPs. The molecular signature achieved an impressive correct classification rate (90%) when expression levels obtained by qRT-PCR from 45 independent formalin-fixed paraffin-embedded (FFPE) SSA/P and HP specimens were used for validation. These results demonstrate the clinical value of the molecular signature.

## Methods

### RNA-seq training data set

The RNA-seq data set used in this study consists of a subset of the NCBI gene expression omnibus (GEO) series with the accession number GSE76987 [39]. We included 10 control left colon (CL), 10 control right colon (CR), 10 microvesicular hyperplastic polyps (MVHPs), and 21 sessile serrated adenoma/polyps (SSA/Ps) specimens with unequivocal diagnoses based on expert gastrointestinal pathology reviews. Raw single-end (SE) RNA-seq reads of 50 base pairs were provided in FASTQ file format from the Illumina HiSeq 2000 platform. To insure high quality reads the fastX-toolkit (version 0.0.13) was employed to discard any read with median Phred score < 30. The surviving sequence reads were aligned to the UCSC hg19 human reference genome using Tophat [40] (version 2.0.12). Tophat aligns RNA-seq reads to mammalian-sized genomes using the high-throughput short read aligner Bowtie [41] (version 2.2.1) and then analyzes the mapping results to identify splice junctions between exons. Cufflinks [40] was used to quantify the abundances of genes, taking into account biases in library preparation protocols. Cufflinks implements a linear statistical model to estimate the assigned abundance to each transcript that explains the observed reads (especially reads originating from a common exon in several isoforms of the same gene) with maximum likelihood. The normalized gene expression values are provided in fragments per kilobase per millions (FPKM) of mapped reads. The log_2_(1 + FPKM) transformation was applied to FPKM values in all analyses.

### Illumina microarrayy testing data set

This data set consists of six normal colon samples, six microvesicular hyperplastic polyps (MVHPs) and six sessile serrated adenomas/polyps (SSA/Ps) [32]. The total RNA was converted to cDNA and modified using the Illumina DASL-HT assay and hybridized to the Illumina HumanHT-12 WG-DASL V4.0 R2 expression beadchip. The biopsies were classified by seven gastrointestinal pathologists who reviewed 109 serrated polyps and identified 60 polyps with consensus. The log_2_-scale of the expression measurements provided under the gene expression omnibus (GEO) accession number GSE43841 was used. Only MVHP and SSA/P samples were considered for the analyses. Illumina probe identifiers were mapped to gene symbol identifiers using the Bioconductor annotation package *illuminaHumanWGDASLv4.db*. Whenever multiple probes were mapped to the same gene, the probe with the largest *t*-statistic between MVHP and SSA/P was selected.

### Affymetrix testing data set

We considered subsets of samples from two GEO data sets, GSE10714 and GSE45270. The total RNA was extracted from 11 patients with hyperplastic polyps (HPs) [36] from GSE10714 and from 6 patients with sessile serrated adenoma/polyps (SSPs) [19] from GSE45270. Genome-wide gene expression profile was evaluated by the HGU133plus2 microarrays from Affymetrix. The background correction, normalization, and probe summarization steps were implemented using the robust multi-array (RMA) method [42] for the combined samples. Probe identifiers were mapped to gene symbol identifiers using the Bioconductor annotation package *hgu133plus2.db*. When multiple probes were mapped to the same gene, the probe with the largest *t*-statistic between the 11 HP samples and the 6 SSA/P samples was selected.

### Biospecimens for independent validation studies

Formalin-fixed paraffin embedded (FFPE) specimens of SSA/Ps (*n* = 21, size range 0.3–3 cm) and HPs (*n* = 24, size range 0.3–0.5 cm) with an unequivocal diagnosis based on the review of at least two independent expert GI pathologists were analyzed. The specimens came from University of Arkansas for Medical Sciences, Little Rock AR Central Arkansas Veterans Healthcare System, Little Rock AR and Cleveland Clinic, Cleveland OH. SSA/Ps were from the right colon (hepatic flexure to cecum) and HPs were from both the left and transverse colon. All samples represented unused de-identified pathologic specimens that were obtained under IRB approval. Total RNA was extracted from six to seven 10 μm slices of FFPE tissues using a RNeasy FFPE kit (Qiagen, Germany) according to the manufacturer’s instructions. The concentration of extracted RNA was determined by Qubit RNA HS assays. Reverse transcription reactions were performed utilizing high capacity RNA-to-cDNA kit (Applied Biosystems, Carlsbad, CA) in 20 μL reactions containing 1 μg of RNA, in compliance with the manufacturer’s protocol.

qPCR was performed with an ABI 7900HT Fast Real-Time PCR System (Applied Biosystems, Carlsbad, CA). With the exception of gene SBSPON, primers for twelve genes were selected from the PrimerBank database [43], and specific primers for SBSPON were purchased from OriGene Technologies (Rockville, MD) (Additional file 1: Table S1). As a control we utilized human 18S ribosomal RNA (Qiagen, Germany). 15 μL reaction mixtures contained 7.5 μL of PowerUp SYBR green 2X master mix (Applied Biosystems, Carlsbad, CA), 0.75 μL of each primer pair (10 μM), and 20 ng of cDNA. The reaction involved initial denaturing for 2 min at 95 °C, followed by 40 cycles of 95 °C for 15 s and 60 °C for 60 s. All analyses were carried out in triplicates.

### Differential expression analysis

Differentially expressed (DE) genes were detected using the returned values from the Cuffdiff2 algorithm [40]. Expressed genes with adjusted *p*-values *P*_adj_ < 0.05 and absolute log_2_ fold change >0.5 were considered DE. *P*-values were controlled for multiple testing using the Benjamini-Hochberg false discovery rate (FDR) method.

### Feature selection step (concordant genes)

**Fig. 1:**
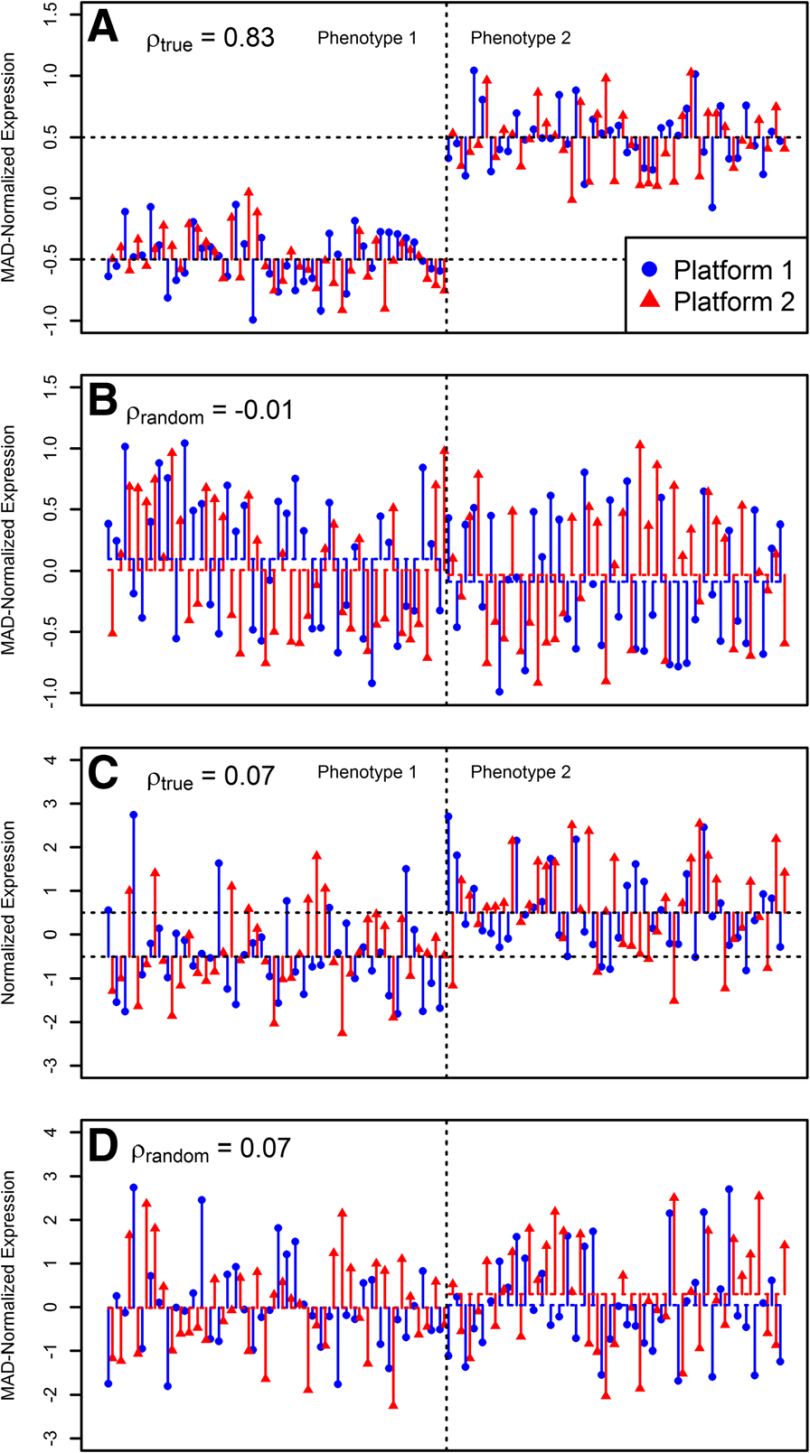
Examples illustrating the new feature selection step. **a** The fold change in both platforms was larger than the within-phenotype variability and the correlation coefficient between platforms (*ρ*_*true*_) was high; **b** when phenotypic labels in part A were randomly resampled, the fold change in both platforms became negligible as compared to the within-phenotype variability and the correlation coefficient between platforms (*ρ*_*random*_) became low. **c** The fold change in both platforms was smaller than the within-phenotype variability and the correlation coefficient between platforms (*ρ*_*true*_) was low; **d** when phenotypic labels in part C were randomly resampled, the correlation coefficient (*ρ*_*random*_) was low

We developed the following algorithm for selecting genes, concordant between platforms:

1. Let matrices *X* = [*X*_1_, …, *X*_*n*_] and *Y* = [*Y*_1_, …, *Y*_*m*_] represent *n*(*m*) *p*-dimensional measurements of gene expression from two platforms. Let *n* = *n*_1_ + *n*_2_, *m* = *m*_1_ + *m*_2_ where *X*(*Y*) has *n*_1_(*m*_1_) samples that belong to phenotype 1 and *n*_2_(*m*_2_) samples that belong to phenotype 2.
2. Sample without replacement from each platform selecting *min*(*n*_1_, *m*_1_) random samples that belong to phenotype 1 and *min*(*n*_2_, *m*_2_) random samples that belong to phenotype 2. Find the Pearson correlation coefficient between the two platforms for each of the *p* genes. These correlations are calculated with actual phenotype labels (*ρ*_*true*_).
3. Sample without replacement from each platform selecting *min*(*n*_1_, *m*_1_) and *min*(*n*_2_, *m*_2_) random samples that belong to any phenotype. Find the Pearson correlation coefficient between the two platforms for each of the *p* genes. These correlations are calculated when samples from both phenotypes are randomly sampled (*ρ*_*random*_).
4. Repeat steps 2 and 3 for a large number of times (we use 10^4^ times) and record the *p* (number of genes) correlation values in each step to estimate the distribution of *ρ*_*true*_ and *ρ*_*random*_ (see Additional file 2: Figure S1). Calculate pooled standard deviation for each gene from the two estimated distributions of *ρ*_*true*_ and *ρ*_*random*_ and use the maximum value *max*(*SD*(*ρ*_*true*_ ∪ *ρ*_*random*_)) for step 5.
5. Use the non-parametric Wilcoxon’s test of means to test the one-sided hypothesis *H*_0_: ${\overline{\rho}}_{true}\le {\overline{\rho}}_{random}+\mathit{\max}\left( SD\left({\rho}_{true}\cup {\rho}_{random}\right)\right)$against the alternative *H*_1_: ${\overline{\rho}}_{true}>{\overline{\rho}}_{random}+\mathit{\max}\left( SD\left({\rho}_{true}\cup {\rho}_{random}\right)\right).$ This test rejects the null hypothesis for genes that are consistently over-expressed in one phenotype under both platforms, especially when the within-phenotype variability is negligible compared to the fold change (see Fig. 1). The term *max*(*SD*(*ρ*_*true*_ ∪ *ρ*_*random*_)) can optionally be multiplied by a constant to increase or decrease the number of genes that rejects the null hypothesis.

### Building the classifier

The shrunken centroid classifier (SCC) works as follows. First, it shrinks each phenotype gene centroids towards the overall centroids and standardizes by the within-phenotype standard deviation of each gene, giving higher weights to genes with stable within-phenotype expression [38]. The centroids of each phenotype deviate from the overall centroids and the deviation is quantified by the absolute standardized deviation. The absolute standardized deviation is compared to a shrinkage threshold and any value smaller than the threshold leads to discarding the corresponding gene from the classification process.

To select the threshold for the centroid shrinkage, a 3-fold cross-validation over a range of 30 threshold values for 100 iterations was performed (R package pamr version 1.55). The threshold returning the minimum mean error with the least number of genes was selected. Within every iteration, genes’ ability to separate between HP and SSA/P samples was assessed by calculating the area under the ROC curve (R package ROCR version 1.0–7) and only genes with AUC > 0.8 were left in the signature. The signature was employed with the SCC to classify independent validation samples as either HPs or SSA/Ps. For a *p*-dimensional validation sample *X* ^*^, the classifier calculates a discriminant score *δ*_*k*_(*X* ^*^) for class *k* and assigns the class with *min*_*k*_(*δ*_*k*_(*X* ^*^)) as the classification decision [38]. Discriminant scores are used to estimate class probabilities (posterior probabilities) as a measure of the certainty of classification decision

$$ {p}_k\left({X}^{\ast}\right)=\frac{e^{-\frac{1}{2}{\delta}_k\left({X}^{\ast}\right)}}{\sum \limits_{m=1}^M{e}^{-\frac{1}{2}{\delta}_m\left({X}^{\ast}\right)}} $$

where *M* is the number of classes [38].

### Classification of independent FFPE samples

Expression levels of 13 genes were estimated relative to a reference level of 18S ribosomal RNA gene, such that larger values represent lower expression levels and smaller values represent higher expression levels (see Additional file 2: Figure S2). Some samples were positively or negatively biased relative to each other (see Additional file 2: Figure S3A). Therefore, raw expression levels were normalized using two steps. First, raw expressions were shifted by their respective sample means or medians to remove any possible positive or negative biases between samples and center expression levels around zero. This step is crucial to reduce technical variation between samples [44]. We tried three options that keep gene ranks in each sample unchanged (arithmetic mean, geometric mean, and median) and noticed no significant difference in the classification results (see Additional file 1: Table S2). We also found that the quantile normalization yielded lower performance (data not shown). Although subtracting the arithmetic or geometric mean showed minor improvement in Additional file 1: Table S2, subtracting the median is recommended when outliers are present in some samples. Expression levels are then multiplied by −1 to let higher expression levels be represented by larger values. Second, the gene-wise MAD normalization was applied such that genes with large fold changes between HPs and SSA/Ps are likely to have positive values under one phenotype and negative values under the other. The normalized expression levels are shown in Additional file 2: Figure S3B. The summary metric (SM) is used to score each sample and each sample is then labeled as HP if SM < 0 and as SSA/P if SM > 0.

Additional file 2: Figures S4 and S5 have shown that the distribution of the MAD-normalized expression and the distribution of SM in one RNA-seq and two microarray data sets were comparable hence the shrunken centroid classifier trained with RNA-seq data can be applied successfully to classify microarray samples. Accurate estimates of the summary metric distribution for each platform allowed proper standardization of the summary metric and hence proper phenotype assignment probability using CLB. While this approach works for high throughput platforms that profile thousands of genes, it is not applicable under typical clinical settings when qRT-PCR is used to profile only a few genes because the distribution of SM is unknown. This is why phenotype assignment probabilities are not available when platforms that profile a few genes (such as small-scale qRT-PCR) are used.

To classify new qRT-PCR samples using our simple approach, the two normalization steps above must be applied. R code implementing the two normalization steps and classifying samples using the summary metric of 13 genes is provided in Additional file 3: Text S1. To apply MAD normalization to real-time qRT-PCR expression levels, multiple samples are necessary to estimate the median expression level for each gene accurately. Therefore we provided the raw qRT-PCR expression levels for the FFPE data set (24 HPs and 21 SSA/Ps) in Additional file 1: Table S3 to allow the normalization of any new qRT-PCR samples. The first normalization step resolve any potential shift biases between the new samples and the samples in Additional file 1: Table S3.

## Results

### Expression analysis

#### Filtering steps

**Fig. 2:**
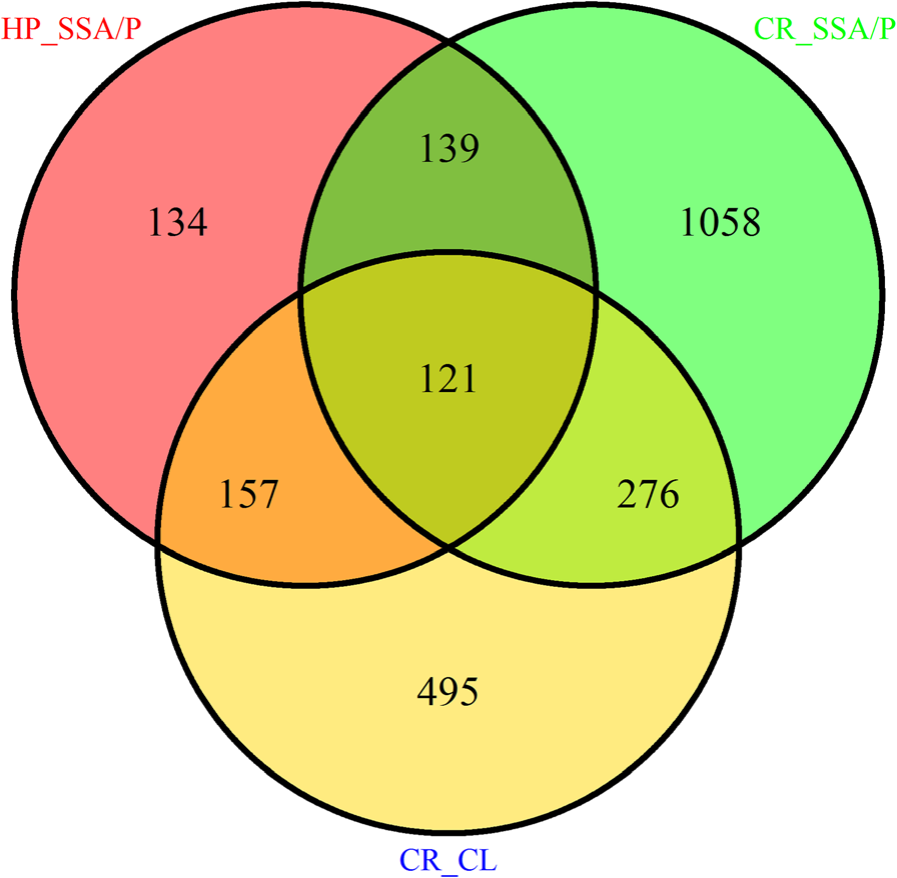
Venn diagram summarizing the DE genes in three comparisons

Genes were called DE if two conditions were met: |log_2_FC| > 0.5 and adjusted *p-*values *P*_adj_ < 0.05 (see Methods for more detail). The intersections of the three comparisons: (1) Control Right (CR) versus Control Left (CL) samples (CR_CL), (2) HP versus SSA/P samples (HP_SSA/P) and (3) CR versus SSA/P samples (CR_SSA/P) are shown in Fig. 2. There were 1049 genes DE between CR and CL samples, and among these genes 157 were also DE between HPs and SSA/Ps and 276 were DE between CR and SSA/P samples. There were 121 genes in the intersection of all three comparisons. With the aim of identifying only genes that reliably differentiate between HPs and SSA/Ps as well as between SSA/Ps and CR samples, we excluded the three aforementioned groups from the further study. The following groups were considered for further analysis: (1) 139 genes that were DE between SSA/Ps and both HP and CR samples (Additional file 1: Table S4), (2) 134 genes, exclusively DE between HPs and SSA/Ps (Additional file 1: Table S5) and (3) 1058 genes, exclusively DE between CR and SSA/P samples (Additional file 1: Table S6). The 121 genes in the intersection of all three comparisons (Additional file 1: Table S7) were excluded for the sake of rigor, i.e. for considering only genes that were DE between different polyp types, without referring to the anatomical location. Although these 121 genes were excluded here, further investigation is needed to assess their importance in differentiating between HPs and SSA/Ps.

**Fig. 3:**
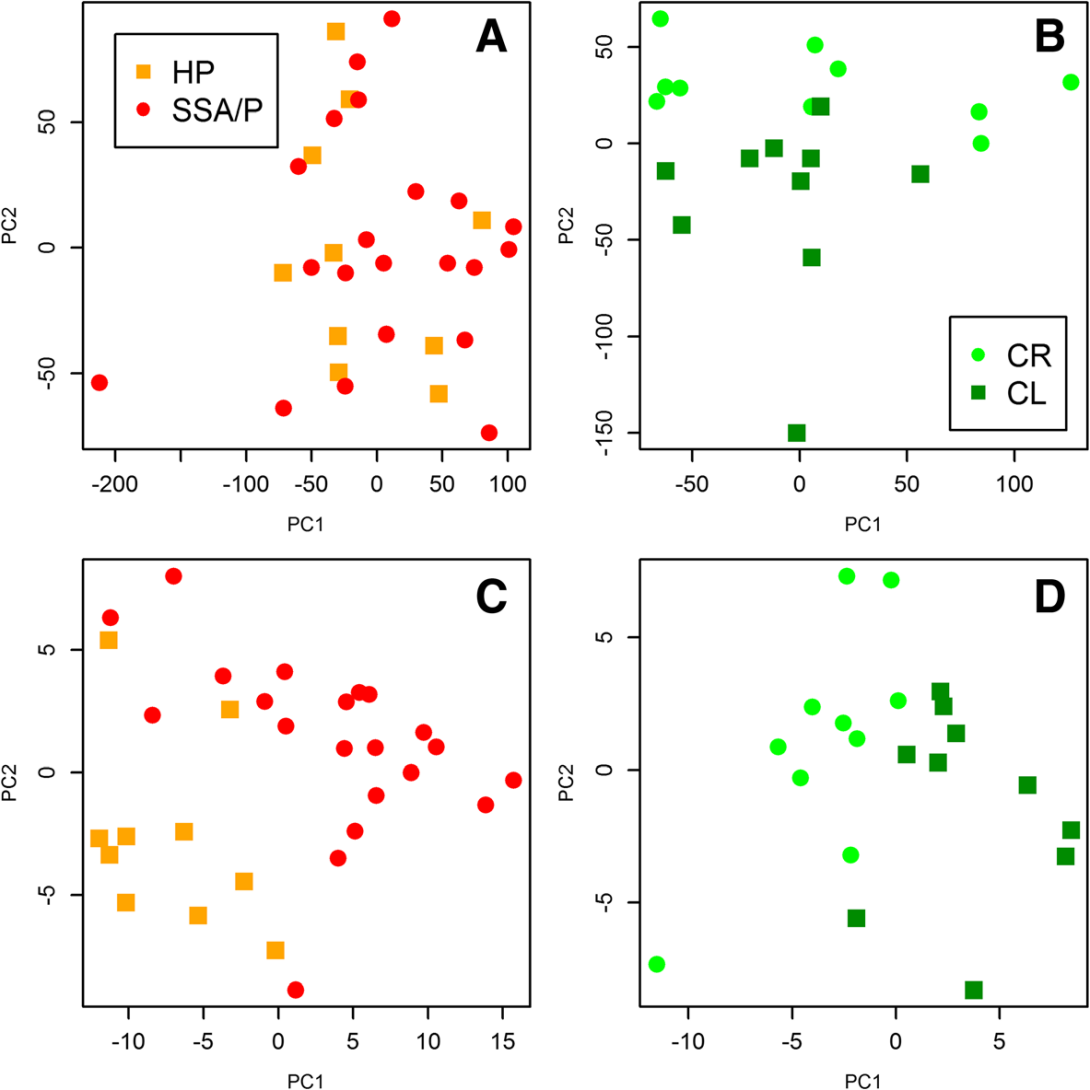
Principle component analysis (PCA) scatter plots. **a** SSA/P and HP samples are not well-separated when all the expressed genes are considered; **b** control right (CR) and control left (CL) samples are well-separated when all the expressed genes are considered; **c** SSA/P and HP samples are well-separated when only the genes differentially expressed between SSA/Ps and both HPs and CRs with the exclusion of genes DE between CR and CL are considered (139 genes); **d** CR and CL samples are well-separated when only the 139 genes in (**c**) are considered

A Principal Component Analysis (PCA) plot illustrating the difficulties in differentiating between SSA/P and HP samples even at the molecular level is presented in Fig. 3. The two groups are clearly intermingled when all expressed genes are included (Fig. 3a) and the separation is much better when genes, DE between HPs and SSA/Ps as well as between SSA/Ps and CR samples are included with the exclusion of genes DE between CR and CL samples (Fig. 3c). Thus, the filtering step allows a more detailed characterization of the differences between HPs and SSA/Ps and improved separation of the two types of polyps.

#### Characteristic differences between SSA/Ps and other samples

To understand more clearly the biological differences between SSA/Ps and other samples we first considered only genes expressed at the same level in HP and CR samples and significantly up- or down-regulated in SSA/Ps. At this step we considered only genes satisfying the following conditions: (1) gene expression level (*e*) satisfied an equation: *e = |*(*CR - HP*)*|*/(*CR + HP* + 0.01) < 0.1 and (2) gene was significantly DE in CR_SSA/P and HP_SSA/P comparisons.

**Fig. 4:**
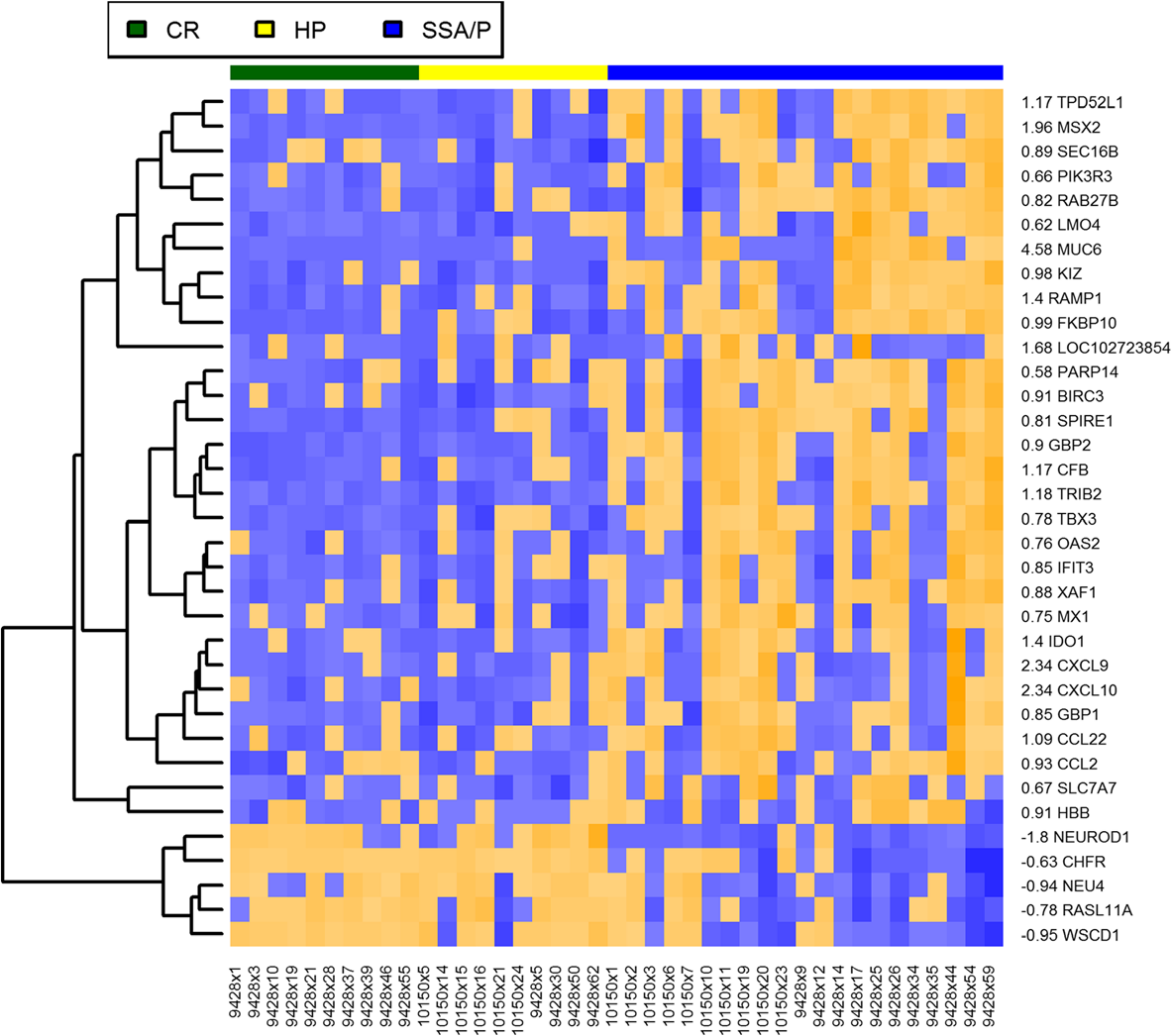
Heatmap of RNA-seq expression data. Hierarchical clustering of CR (green), HP (yellow) and SSA/Ps (blue) biopsies (columns) and differentially expressed genes (rows). Only genes that were expressed at the same level in HP and CR samples but significantly up- or down-regulated in SSA/Ps are shown. Down-regulated and up-regulated genes in SSA/Ps are indicated in blue and orange colors, respectively. The log_2_(SSA/P / HP) is shown on the left side of gene names

There were only five genes, down-regulated in SSA/Ps and expressed at the same level in HPs and CRs (Fig. 4). Two of them regulate cell differentiation and proliferation: NEUROD1 (neuronal differentiation 1) is involved in enteroendocrine cell differentiation [45] and CHFR (checkpoint with forkhead associated and RING Finger) is an early mitotic checkpoint regulator that delays transition to metaphase in response to mitotic stress. CHFR has been found to be frequently inactivated in many malignancies by promoter methylation [46, 47], in particular, in microsatellite stable and BRAF wild-type CRCs stage II [48]. NEU4, another down-regulated gene, maintains normal mucosa and its down-regulation was suggested to contribute to invasive properties of colon cancers [49]. Other down-regulated genes are RASL11A (regulates translation and transcription) and WSCD1 (WSC domain containing 1, poorly characterized).

Twenty out of thirty genes, up-regulated in SSA/Ps and expressed at the same level in CR and HP samples, were found to be interferon-regulated (IR) [50]. In addition to modulating innate immune response, interferons regulate a large variety of cellular functions, such as cell proliferation, differentiation, as well as play important roles in inflammatory diseases [51] and anti-tumor response [52]. These twenty genes were represented by (1) genes, involved in the epithelial-mesenchymal transition (EMT): PIK3R3 [53], RAB27B [54] and MSX2 [55]; (2) classical IR genes: GBP2, CFB, TRIB2, TBX3, OAS2, IFIT3, XAF1, MX1, IDO1, CXCL9,CXCL10, GBP1, CCL22, CCL2; (3) genes, not conventionally considered IR: RAMP1, PARP14 and TPD52L1.

Among these twenty genes there were three, especially interesting in the context of risk of SSA/Ps progressing to cancer. Indoleamine 2,3-dioxygenase 1 (IDO1) has attracted considerable attention recently because of its immune-modulatory role besides the degradation of tryptophan. IDO regulates T cell activity by reducing the local concentration of tryptophan and increasing the production of its metabolites that suppress T lymphocytes proliferation and induce apoptosis [56–58]. Because most human tumors constitutively express IDO [58], the idea that IDO inhibitors may reverse immune suppression, associated with tumor growth, is very attractive for immunotherapy and a competitive inhibitor for IDO (l-MT) is currently in clinical trials [59]. IDO1 was 2.7 times up-regulated in SSA/Ps as compared to HP, CR samples. PIK3R3, an isoform of class IA phosphoinositide 3-kinase (PI3K), that specifically interacts with cell proliferation regulators and promotes metastasis and EMT in colorectal cancer [53], was also up-regulated in SSA/Ps. PARP14 promotes aerobic glycolysis or the Warburg effect, used by the majority of tumor cells, by inhibiting pro-apoptotic kinase JNK1 [60]. Immunosuppressive state, the shift toward aerobic glycolysis and the EMT, are all considered the major hallmarks of cancer [35]. While these three genes are only infinitesimal parts of the invasive cascades, their up-regulation points toward how SSA/Ps may progress to cancer.

Several interferon regulated (IR) genes reported here have been also found to be up-regulated in a number of malignancies (including CRCs). For example, RAB27B was expressed at a high level and is a special member of the small GTPase Rab family regulating exocytosis which has been associated with a poor prognosis in patients with CRC [61]. Increased expression of RAB27B has been shown to predict a poor outcome in patients with breast cancer [62]. The suggested mechanism by which Rab27b stimulates invasive tumor growth includes regulation of the heat shock HSP90α protein and the indirect induction of MMP-2, a protease that requires an association with extracellular HSP90α for its activity to accelerate the degradation of extracellular matrix [62]. The transcription factor TBX3 (T-box 3), which plays an important role in embryonic development, was also up-regulated in SSA/Ps. Previously it was suggested that TBX3 promotes an invasive cancer phenotype [63] and more recently it was also shown that increased expression of TBX3 was associated with a poor prognosis in CRC patients [64]. The transcriptional co-regulator LIM-only protein 4 (LMO4) has been associated with poor prognosis and is overexpressed in about 60% of all human breast tumors and has been shown to increase cell proliferation and migration [65]. LMO4 was up-regulated in SSA/Ps. Tumor protein D52-like proteins (TPD52) are small proteins that were first identified in breast cancer, are overexpressed in many other cancers, but remain poorly characterized [66]. TPD52L1, member of the family, was upregulated in SSA/Ps.

Besides the twenty IR genes, there were other interesting genes up-regulated in SSA/Ps and expressed at the same level in CR and HP samples. MUC6 (mucin 6) was the most highly up-regulated gene and has been previously suggested as a candidate biomarker for SSA/Ps [67, 68] but later was found to be not specific enough to reliably differentiate SSA/Ps from HPs [69]. KIZ (kizuna centrosomal protein) is a gene that is critical for the establishment of robust mitotic centrosome architecture and proper chromosome segregation at mitosis [70]. While depletion of KIZ results in multipolar spindles, how up-regulation of KIZ affects mitosis is unknown. SPIRE1, an actin organizer, was recently found to contribute to invadosome functions by speeding up extracellular matrix lysis while overexpressed [71].

One of the limitations of studying differentially expressed genes one gene at a time is that it does not allow a systems-level view of global changes in expression and co-expression patterns between phenotypes. Thus, we sought to identify all pathways that were significantly up- or down-regulated, as well as differentially co-expressed between SSA/Ps and HP, CR samples. Pathways were presented by all gene ontology (GO) [72] terms from C5 collection of gene sets in MSigDB [73].

#### Pathways, differentially expressed between SSA/Ps and HP, CR samples

To find pathways, significantly up- or down-regulated we applied ROAST, a parametric multivariate rotation gene set test [74]. ROAST uses the framework of linear models and tests whether for all genes in a pathway, a particular contrast of the coefficients is non-zero [74]. It can account for correlations between genes and has the flexibility of using different alternative hypotheses, testing whether the direction of changes for a gene in a pathway is *up*, *down* or *mixed* (up or down) [74]. We selected only pathways where genes were significantly up- or down-regulated (FDR < 0.05). There were fifteen pathways, significantly up-regulated in SSA/Ps as compared to HP, CR samples (Table 1). In agreement with the pattern found for individual genes, two out of the fifteen pathways were ‘Inflammatory response’ and ‘Immunological synapse’ (Table 1). GO term ‘Extracellular structure organization and biogenesis’ overlaps with two KEGG pathways: ‘KEGG focal adhesion’ and ‘KEGG ECM receptor interaction’. Overexpression of these pathways as well as ‘Cell adhesion’ (two pathways) category might indicate changes in cell motility and migration ability in SSA/Ps as compared to HP, CR samples. Up-regulation of ‘Cell growth and death’ (two pathways) category suggests increased cellular proliferation in SSA/Ps.

**Table 1:** Up-regulated pathways (GO categories)

Category	Pathway	FDR^a^
Cell adhesion		
	CALCIUM INDEPENDENT CELL CELL ADHESION	0.022
	CELL SUBSTRATE ADHERENS JUNCTION	0.042
Cell growth and death		
	CELL STRUCTURE DISASSEMBLY DURING APOPTOSIS	0.033
	POSITIVE REGULATION OF CELL PROLIFERATION	0.033
Immune system		
	INFLAMMATORY RESPONSE	0.033
	IMMUNOLOGICAL SYNAPSE	0.045
Signal transduction		
	POSITIVE REGULATION OF SECRETION	0.045
	G PROTEIN COUPLED RECEPTOR PROTEIN SIGNALING	0.042
	SECOND MESSENGER MEDIATED SIGNALING	0.045
Metabolism		
	AROMATIC COMPOUND METABOLIC PROCESS	0.022
	HETEROCYCLE METABOLIC PROCESS	0.022
Differentiation		
	CELLULAR MORPHOGENESIS DURING DIFFERENTIATION	0.045
Cellular component organization		
	EXTRACELLULAR STRUCTURE ORGANIZATION AND BIOGENESIS	0.042
Neuron development		
	AXONOGENESIS	0.042
	NEURITE DEVELOPMENT	0.045

^a^FDR: False Discovery Rate

There was only one pathway down-regulated in SSA/Ps as compared to HP, CR samples, namely ‘Transmembrane receptor protein serine threonine kinase signaling pathways’ (FDR < 0.05). The pathway generates a series of molecular signals as a consequence of a transmembrane receptor serine/threonine kinase binding to its ligand and regulates fundamental cell processes such as proliferation, differentiation, death, cytoskeletal organization, adhesion and migration [75]. For this pathway, one of the most significantly down-regulated genes was HIPK2 (homeodomain interacting protein kinase 2). HIPK2 interacts with many transcription factors including p53 and is a tumor suppressor that regulates cell-cycle checkpoint activation and apoptosis. Therefore, its down-regulation may contribute to up-regulation of the ‘Positive regulation of cell proliferation’ pathway. However, given that ‘Transmembrane receptor protein serine threonine kinase signaling pathways’ regulates many fundamental cellular processes, its main downstream targets in the case of SSA/Ps require further study.

#### Pathways, differentially co-expressed between SSA/Ps and HP, CR samples

To find pathways that were differentially co-expressed we applied an approach that assesses multivariate changes in the gene co-expression network between two conditions, the Gene Sets Net Correlations Analysis (GSNCA) [76], as implemented in the Bioconductor package GSAR [77]. GSNCA tests the hypothesis that the co-expression network of a pathway did not change between two conditions. In addition, for each condition it builds a core of co-expression network, using the most highly correlated genes, and finds a ‘hub’ gene, defined as the one, with the highest correlations with the other genes in a pathway (see [76] for more detail). In other words, hub genes are the most ‘influential’ genes in a pathway. When hub genes in a pathway are different between phenotypes, it points toward regulatory changes in a pathway dynamic.

There were seven pathways significantly differentially co-expressed between SSA/Ps and CR, HP samples (*P* < 0.05). Five out of seven were pathways regulating homologous and non-homologous recombination, DNA replication, GTPase activities and proteins targeting towards a membrane using signals contained within the protein (Additional file 2: Figures S6-S10). For all five pathways, hub genes were different between HPs and SSA/Ps, with a shift in SSA/Ps toward hub genes related to genomic instability. For example, for ‘Meiosis I’ and ‘Meiotic recombination’ pathways, hub genes were RAD51 and MRE11A in HPs and SSA/Ps, respectively. Both proteins are involved in the homologous recombination and repair of DNA double strand breaks. MRE11A also participates in alternative end-joining (A-EJ), an important pathways in the formation of chromosomal translocations [78]. The shift from RAD51 to MRE11A in SSA/Ps might indicate an increased genomic instability, the key change in all cancer cells [35].

**Fig. 5:**
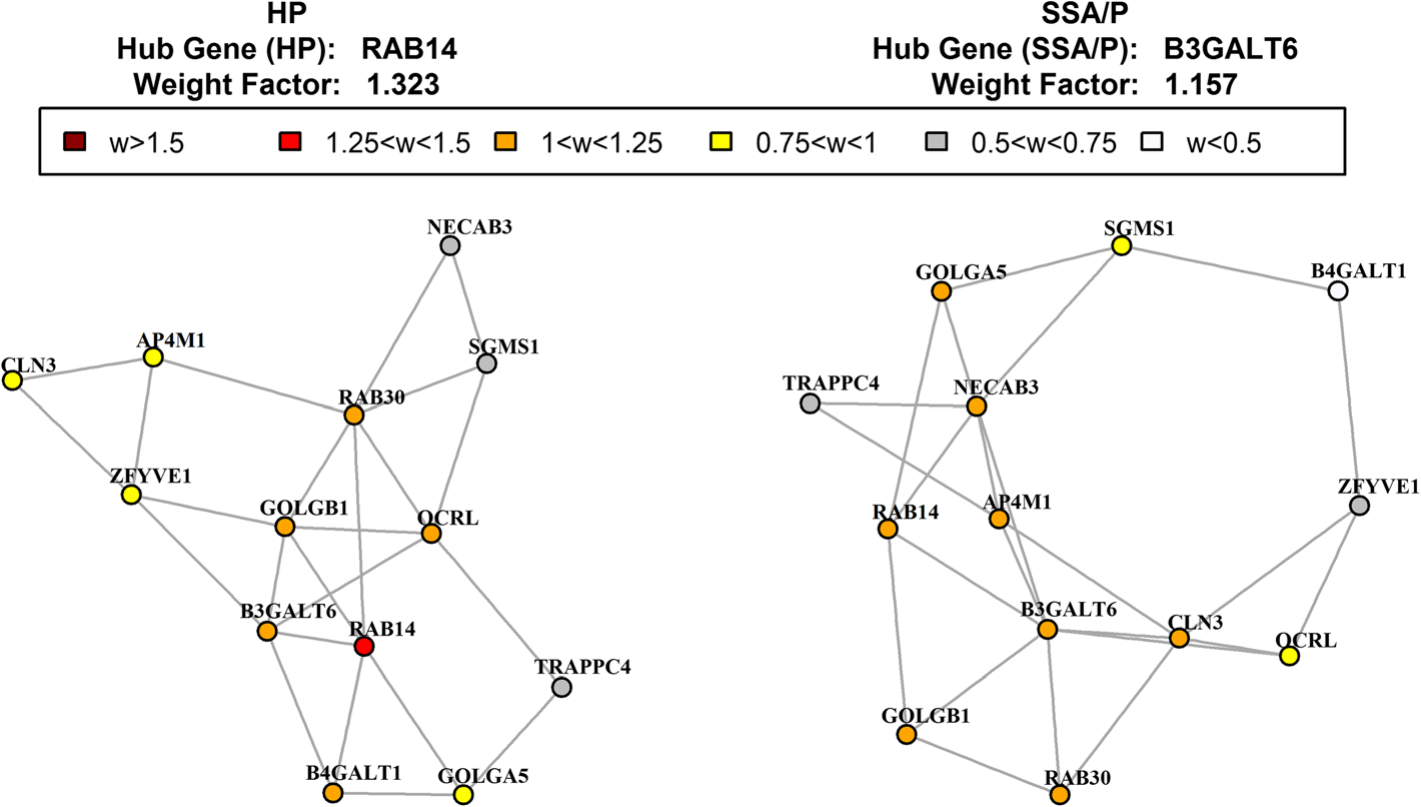
MST2 of the ‘Golgi stack’ gene set from the C5 collection of MSigDB. This gene set was detected by GSNCA (*P* < 0.05) in both comparisons: HPs versus SSA/Ps and CRs versus SSA/Ps

For ‘Golgi stack’ pathway, the shift of hub genes was associated with the well-known phenotypic difference between HPs and SSA/Ps (Fig. 5). The hub gene in HP was RAB14, low molecular mass GTPase that is involved in intracellular membrane trafficking and cell-cell adhesion. The hub gene in SSA/Ps was B3GALT6, a beta-1,3-galactosyltransferase, required for glycosaminoglycan (mucopolysaccharides) synthesis, including mucin. The presence of abundant surface mucin is the conventional colonoscopic characteristic of SSA/Ps [79]. For ‘Hormone activity’ in HP the hub gene was IGF1, the insulin-like growth factor that promotes cell proliferation and inhibits apoptosis, stimulates glucose transport in cells and enhances glucose uptake (Additional file 2: Figure S11). In SSA/P, the hub gene was PYY, encoding a member of the neuropeptide Y (NPY) family of peptides. This gut peptide plays important roles in energy and glucose homeostasis [80], in regulating gastrointestinal motility and absorption of water and electrolytes and has been associated with several gastrointestinal diseases [81]. Its role in SSA/Ps, if any, remains to be defined.

Based on the analysis of individual genes and differentially expressed and co-expressed pathways SSA/Ps difference from HP, CR samples involves: (1) up-regulation of IR genes, EMT genes and genes previously associated with the invasive cancer phenotype; (2) up-regulation of pathways, implicated in proliferation, inflammation, cell-cell adhesion and down-regulation of serine threonine kinase signaling pathway; (3) de-regulation of a set of pathways regulating cell division, protein trafficking and kinase activities.

Given the complexity of the molecular processes underlying SSA/Ps, involving hundreds of differentially expressed genes and many pathways, for the practical purpose of readily distinguishing SSA/Ps from HPs we developed a platform-independent molecular classifier with low classification error rate (see below).

### Molecular classifiers

Typically, the development of molecular classifiers consists of the following steps: feature selection, model selection, training, estimation of the classification error rate [25], with every step potentially leading to an inflated performance estimate [20]. The systematic errors in classifier development, such as inappropriate applications of cross-validation for classifiers’ training and testing, are usually the first to blame for poor generalizability (high error rate on independent data sets) [20, 82, 83]. Poor generalizability is further emphasized when the training and independent test data are obtained using different platforms, e.g. different microarray platforms, or microarrays and RNA-seq. To avoid such errors we developed a new feature selection step identifying the genes, most concordant between different platforms. After the new feature selection step was implemented, we trained a classifier on RNA-seq data and further tested it on two independent microarray data sets (testing sets, see Methods for more details). Identifiers from different platforms were mapped to gene symbols and only genes that were expressed in RNA-seq data and present on both microarray platforms were considered (Additional file 1: Table S8).

#### Feature normalization

For classifier development 139 genes, DE between SSA/Ps and HP, CR samples (Additional file 1: Table S4) were considered. Gene expressions for both RNA-seq and microarray platforms were normalized to a common range by subtracting the median absolute deviation (MAD) [19] from each gene’s expression. Hence, gene expressions were centered around zero and genes with large fold changes between two phenotypes had positive expressions under one phenotype and negative expressions under the other. Genes with the small variability were filtered out (MAD < 0.1). Finally, only the genes expressed in all three platforms (117 genes) were considered for further classifier design steps.

#### Feature selection step

Selecting only genes (features) with high concordance between platforms is crucial to design a platform-independent classifier. Platform-independent classifier, trained using one platform, should have low classification error rate while being tested using other platform. Here, to assess genes concordance between platforms, we developed a new non-parametric test (see Methods for details). The test identified genes, robustly differentiating two phenotypes under different platforms, the best candidates for an inter-platform signature. Previously, the concordance between platforms has been measured by the correlation between mean expressions or fold changes [84, 85] or by intersection between lists of DE genes [86, 87].

Consider two distributions: (1) correlation coefficients for all genes between two platforms, preserving phenotypic labels (*ρ*_*true*_) and (2) correlation coefficients for all genes between two platforms, randomly resampling phenotypic labels (*ρ*_*random*_). Additional file 2: Figure S1 presents the distributions of *ρ*_*true*_ and *ρ*_*random*_ when the HP and SSA/P samples from the RNA-seq training data were compared with the Illumina and Affymetrix data sets. Some genes had higher correlations when phenotypic labels were preserved, compared to when they were randomly resampled, introducing negative skewness to the distribution of *ρ*_*true*_ (see Additional file 2: Figure S1). These genes showed higher correlation between platforms than by chance, illustrated by the case when phenotypic labels were randomly resampled. These genes were our candidate concordant genes. More formally, to identify concordant genes we tested the null hypothesis *H*_*0*_: ${\overline{\rho}}_{true}\le {\overline{\rho}}_{random}+\mathit{\max}\left( SD\left({\rho}_{true}\cup {\rho}_{random}\right)\right)$.

Figure 1 illustrates how the test works using two examples of typical MAD-normalized gene expressions in two platforms. In one example, forty observations were sampled from two normal distributions *N*(0.5, 0.25) and *N*(−0.5, 0.25), representing different phenotypes. In this example, the fold change in both platforms was larger than the within-phenotype variability (Fig. 1a) and the correlation coefficient between platforms (*ρ*_*true*_) was high. When phenotypic labels were randomly resampled, the fold change in both platforms became negligible as compared to the within-phenotype variability (Fig. 1b) and the correlation coefficient between platforms (*ρ*_*random*_) became low. In another example, forty observations were sampled from two normal distributions *N*(0.5, 1) and *N*(−0.5, 1), again representing different phenotypes. However, in this example, the fold change in both platforms was smaller than the within-phenotype variability (Fig. 1c, d) and the correlation coefficient between platforms was low when phenotypic labels were either preserved or randomly resampled. Although the fold change between phenotypes was the same in both examples (*log*_2_ *FC* = 1), Pearson correlation coefficient between expressions in two platforms preserving phenotypic labels (*ρ*_*true*_) was higher in case A compared to case C because of the lower within-phenotype variability. Randomly resampling phenotypic labels led, expectedly, to much lower correlations between two platforms (*ρ*_*random*_) (Fig. 1b, d). Accordingly, *ρ*_*true*_ > *ρ*_*random*_ in the first example (Fig. 1a, b) but not in the second (Fig. 1c, d). Taking average correlation between platforms, for a large number of iterations, *H*_*0*_ will be rejected for the first example (Fig. 1a, b) but not for the second (Fig. 1c, d). The material and methods section summarizes the steps of the proposed test.

The test was used to find genes with high concordance between RNA-seq and Illumina platforms (23 genes detected), RNA-seq and Affymetrix platforms (20 genes detected), and between RNA-seq and both Illumina and Affymetrix platforms (16 genes detected). Only genes, detected by the Wilcoxon’s test at *P* < 0.05 were considered. The values of the term *max*(*SD*(*ρ*_*true*_ ∪ *ρ*_*random*_) were 0.41 and 0.39 when RNA-seq data was compared with Illumina and Affymetrix data sets, respectively.

#### Classifier design and gene signatures

The model selection step provides a great flexibility because there are many machine learning algorithms available for classification purposes. We selected the nearest shrunken centroid classifier (SCC) [38], because it was successfully used before for developing many microarray-based classifiers, in particular a prognostic classifier in CRCs [19]. To select the threshold value that returns the minimum mean error with the least number of genes, we performed a 3-fold cross-validation over a range of threshold values for 100 iterations.

Training the classifier using the RNA-seq data set and considering only the genes with high concordance with the Illumina, Affymetrix, and both platforms yielded three signatures of 18, 16, and 13 genes (see Table 2). The 18 and 16 gene signatures resulted in zero (out of 12 Illumina samples) and three (out of 17 Affymetrix samples) errors. Classification errors did not change when the 13 gene signature was used instead. Hence we considered these 13 genes as the smallest successful signature for both Illumina and Affymetrix platforms. The samples in the Illumina data set were identified as belonging to SSA/Ps or HPs by gastrointestinal pathologists based on a higher stringency criterion [32] than what has been done for the samples in the Affymetrix data set. It is therefore no surprise that there was less ambiguity in classifying the Illumina samples. Although the Illumina samples were acquired by a different platform compared to the training RNA-seq data set, they were classified without errors. Aside from the stringent criterion in assigning phenotype labels for Illumina samples, this result could be due to the higher resolution in quantifying gene expression by the RNA-seq platform.

**Table 2:** Performance of the nearest SCC classifying independent SSA/P and HP microarray samples using three signatures

Platforms	Concordant genes	Signature size	Signature	Illum.^a^ errors	Affy.^b^ errors
Training: RNA-seq Testing: Illumina	C4BPA,CEMIP,CHGA,CLDN1,CPE,DPP10,FSIP2,GRAMD1B,GRIN2D,IL2RG,KIZ,KLK7,MEGF6,MYCN,NTRK2,PLA2G16,RAMP1,SBSPON,SEMG1,SLC7A9,SPIRE1,TM4SF4	18	C4BPA,CHGA,CLDN1,CPE,DPP10,GRAMD1B,GRIN2D,KIZ,KLK7,MEGF6,MYCN,NTRK2,PLA2G16,SBSPON,SEMG1,SLC7A9,SPIRE1,TM4SF4	0	–
Training: RNA-seq Testing: Affymetrix	CLDN1,FOXD1,IDO1,IL2RG,KIZ,LMO4,MEGF6,NTRK2,PIK3R3,PLA2G16,PRUNE2,PTAFR,SBSPON,SEMG1,SLC7A9,SPIRE1,TACSTD2,TPD52L1,TRIB2,ZIC2	16	CLDN1,FOXD1,KIZ,MEGF6,NTRK2,PIK3R3,PLA2G16,PRUNE2,PTAFR,SBSPON,SEMG1,SLC7A9,SPIRE1,TACSTD2,TPD52L1,TRIB2	–	3
Training: RNA-seq Testing: Illumina and Affymetrix	CHFR,CHGA,CLDN1,IL2RG,KIZ,MEGF6,NTRK2,PLA2G16,PTAFR,SBSPON,SEMG1,SLC7A9,SPIRE1,TACSTD2,VSIG1,ZIC2	13	CHFR,CHGA,CLDN1,KIZ,MEGF6,NTRK2,PLA2G16,PTAFR,SBSPON,SEMG1,SLC7A9,SPIRE1,TACSTD2	0	3

^a^Illumina microarrays

^b^Affymetrix HGU133plus2 microarrays

#### Smallest successful signature

The genes, included in the smallest signature (13 genes) were on the average approximately four folds up- (down-) regulated between SSA/Ps and HPs (Table 3). The average absolute fold change considering all the 14,006 expressed genes in the RNA-seq training data set was 1.27. There were three down- and ten up-regulated genes in SSA/Ps, involved in several molecular processes that we have discussed earlier. Down-regulated genes included NTRK2 (neurotrophic tyrosine kinase receptor, type 2), CHFR (negative regulator of cell cycle checkpoint) and CHGA (chromogranin A, endocrine marker). NTRK2 controls the signaling cascade that mainly regulates cells growth and survival.

**Table 3:** Genes, included in the smallest 13 gene expression signature of SSA/Ps

Gene	log_2_FC	FC	Description
SLC7A9	3.22	9.34	Solute carrier family 7 member 9
SEMG1	2.95	7.72	Semenogelin I
MEGF6	2.66	6.34	Multiple EGF like domains 6
TACSTD2	1.93	3.82	Tumor-associated calcium signal transducer 2
CLDN1	1.85	3.59	Claudin 1
SBSPON	1.23	2.35	Somatomedin B and thrombospondin type 1 domain containing
PLA2G16	1.18	2.27	Phospholipase A2 group XVI
PTAFR	1.08	2.11	Platelet activating factor receptor
KIZ	0.98	1.98	Kizuna centrosomal protein
SPIRE1	0.82	1.76	Spire type actin nucleation factor 1
CHFR	−0.62	0.65	Checkpoint with forkhead and ring finger domains, E3 ubiquitin protein ligase
CHGA	−1.63	0.32	Chromogranin A
NTRK2	−2.32	0.20	Neurotrophic tyrosine kinase, receptor, type 2

Up-regulated genes included several genes (SLC7A9, SEMG1, SBSPON and MEGF6) that were not well functionally characterized (except SLC7A9, a marker for cystinuria) and are not discussed here. Two genes (KIZ and SPIRE1) were among the genes up-regulated in SSA/Ps and equally down-regulated in HP, CR samples (Fig. 4). TROP-2 (TACSTD2, tumor-associated calcium signal transducer 2) is a cell-surface transmembrane glycoprotein overexpressed in many epithelial tumors. TROP-2 was suggested as a biomarker to determine the clinical prognosis and as a potential therapeutic target in colon cancer [88, 89] and an antibody-drug conjugate targeting TROP-2 is currently in phase II clinical trials [90]. Claudin-1 (CLDN1, tight junction protein) was also up-regulated. Specifically, Claudin-1 has been suggested to be involved in the regulation of colorectal cancer progression by up-regulating Notch- and Wnt-signaling and mucosal inflammation [91]. In addition, CLDN1 was also associated with liver metastasis of CRC [92]. PLA2G16 phospholipase was also up-regulated and its up-regulation may be a signal of gain-of-function activities of mutant p53 that is required for metastasis [93]. Finally, PTAFR, platelet activating factor receptor, was found to stimulate EMT by activating STAT3 cascade [94].

In sum, the up-regulated signature genes included those previously associated with invasive cell activities (CLDN1, PLA2G16, PTAFR, SPIRE1), spindle formation (KIZ) while down-regulated genes included checkpoints controlling cell growth (CHFR, NTRK2).

#### Summary metric with class probability

The ultimate goal of building a classifier and finding genes signature is to use the signature in clinical practice for diagnostic and prognostic purposes. Here, we developed a simple procedure that uses the signatures in Table 2 to classify new samples as either HP or SSA/P and provides a class probability for the decision. The mean of the MAD-normalized expression of the genes in the signature was used as a summary metric (SM). Since most of the genes in the signatures in Table 2 were over-expressed in SSA/Ps, SM > 0 for SSA/P samples and SM < 0 for HP samples. Before calculating the mean expression, the signs of the expressions of the few genes that were over-expressed in HP were inverted. This step increased the magnitude of the mean regardless of its sign. There were only three genes over-expressed in HP in the 13-gene signature (CHFR, CHGA and NTRK2), one in the 16-gene Affymetrix signature (NTRK2), and four in the 18-gene Illumina signature (CHGA, CPE, DPP10, and NTRK2). The class assignment (HP or SSA/P) depends simply on the sign of the mean expression.

**Fig. 6:**
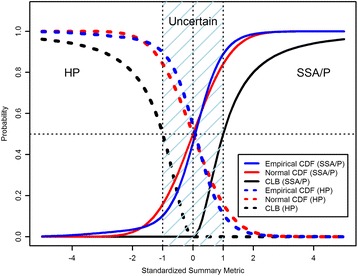
The probability of an assigned SSA/P (HP) class is the cumulative distribution function CDF(SM) (1-CDF(SM)) of the empirical distribution of SM after standardization. The empirical approach can also be substituted by the normal approximation of SM. Since both approaches have limitations, the Cantelli lower bound (CLB) is used as a conservative probability assignment for the SM score

MAD-normalized gene expressions had approximately a Laplace-like distribution (Additional file 2: Figure S4) and the SM distribution was approximately normal (Additional file 2: Figure S5). According to the central-limit theorem, the SM distribution should be normal, especially for signatures with a large number of genes *p* ≥ 30 (Additional file 2: Figure S5). The normal approximation is still valid when the signature size *p* < 30 if the population is not too different from a normal distribution [95]. There are several ways of assigning a class probability to a new sample using training RNA-seq data set as a reference. The distribution of SM can be estimated by calculating SMs for many random signatures of the same size as the signature in use. The probability of an assigned SSA/P (or HP) class is the cumulative distribution function CDF(SM) (1-CDF(SM)) of the empirical distribution of SM after standardization (Fig. 6). Another possibility is to use the normal approximation of SM (Fig. 6). The first approach is impaired by the possible differences in the distribution of SM between different platforms. For example, applying MAD normalization to the log_2_-scale FPKM RNA-seq data yielded SM with negative tail that extended beyond the corresponding tail in microarray data (Additional file 2: Figure S5). The second approach is impaired by deviation from normality especially for very small signatures. Generally, the distribution of SM was normal-like with higher kurtosis for small signatures. While the distribution of SM had kurtosis ≈8 and 4 for RNA-seq and microarray data, respectively (using 15 genes in a signature), the kurtosis of a standard normal distribution is 3.

Due to the potential difficulties in fitting an exact distribution to SM another solution was found. A lower bound for *P*(*X* ≥ *SM*) as the probability for an assigned SSA/P class and *P*(*X* ≤ -*SM*) as the probability for an assigned HP can be estimated using Cantelli’s inequality (also known as one-sided Tchebycheff’s inequality) [96]. Cantelli’s inequality estimates an upper bound for the probability that observations from some distribution are bigger than or smaller than their average:

$$ P\left(X-\mu \le a\right)= CDF\left(\mu +a\right)\ge 1-\frac{\sigma^2}{\sigma^2+{a}^2},a\ge 0 $$

$$ P\left(X-\mu \le a\right)= CDF\left(\mu +a\right)\le \frac{\sigma^2}{\sigma^2+{a}^2},a<0 $$

We either choose *a* = SM and *σ* = 0.14 (which happened to be a standard deviation of SM in all three platforms when the number of genes is 15), or choose *a* = standardized SM and *σ* = 1. Figure 6 presents the Cantelli lower bound (CLB) for SSA/P and HP probabilities. When *SM* ∈ [−*σ*, *σ*] (or *SM*_*standardized*_ ∈ [−1, 1]) the probability of class assignment is zero for one class and <50% for the other, therefore no probability was assigned (*Uncertain* zone*,* Fig. 6). To avoid false positive the probability was assigned if and only if Cantelli lower bound of SM was >0.5. The results of classifying samples in the Illumina and Affymetrix data sets using the summary metric and the class probability assigned to each decision are presented in Additional file 1: Tables S9, S10 and S11. For comparison, the class probabilities obtained using the empirical approach, normal approximation, and the SCC (independent of SM) are also shown. Standardized SM and *σ* = 1 were used. When the Affymetrix samples were classified using the 16-gene signature, 2 of the 3 misclassified HP samples by SCC were deemed uncertain by CLB while assigned *P*(SSA/P) of 75% and 94% by SCC (Additional file 1: Table S10).

### Independent validation and clinical diagnostic tool

To further validate the accuracy of the 13 gene molecular signature and demonstrate its diagnostic value in clinically relevant settings, we obtained expression levels from 45 (24 HPs and 21 SSA/Ps) independent FFPE SSA/P and HP samples with qRT-PCR (see Methods). By simply applying proper normalization and summarizing expression levels using the summary metric (see Methods) the 13 gene molecular signature correctly classified 90% of the independent FFPE samples (Additional file 1: Table S2). Additional file 2: Figure S12 shows the scatter plot of the first and second principle components of normalized expression levels. The 13 gene molecular signature indeed placed HP and SSA/P independent FFPE samples in two well-separated clusters. This approach is simple and relies on the ability of the combined 13 genes to accurately distinguish between HP and SSA/P, rather than relying on a complex classifier. The steps required to apply this simple approach as a clinical diagnostic tool to new FFPE specimens are summarized in the Methods. Notably, the gene signature for SSA/Ps found using RNA-seq data from fresh specimens achieved a remarkably correct classification rate despite issues with RNA degradation in archived FFPE samples.

## Discussion

Conventionally, SSA/Ps are distinguished from HPs primarily based on histopathological morphology [97]. Because HPs can have similar morphological features a significant error rate of classifying SSA/Ps as HPs can occur, especially if expert gastrointestinal pathologists are not available. This clinical challenge was the driver of this study, which aimed to better differentiate SSA/Ps from HPs and develop a platform independent molecular classifier for SSA/Ps. Previously, the differences between SSA/Ps and HPs were considered mostly at the level of individual genes. The genes DE between SSA/Ps and CR (or HP) samples (MUC17 [33], TFF1 and CTSE [31], SLIT2 [98]) were also found in the present analysis. However, we found that these genes were also DE between CR and CL samples, so their association with SSA/Ps is uncertain. Among other potential biomarkers for SSA/Ps (ANXA10, FABP6 and TTF2) ANXA10 was found to be significantly DE between HPs and SSA/Ps and TFF2 was found to be significantly DE between SSA/Ps, HPs, and CR specimens (Additional file 1: Table S5 and Additional file 3: Text S1) [32]. However, FABP6 was not significantly DE between SSA/Ps, HPs, and CR specimens.

A systems-level view of the differences between HPs and SSA/Ps obtained by analyzing different functional units (genes and pathways) and different regulatory relationships (differential expression and co-expression) in the data set provided new insights into the biology of SSA/Ps. When we considered only genes significantly up- or down-regulated in SSA/Ps and expressed at the same level in HPs and CR specimens two thirds of the up-regulated genes were interferon-regulated genes, including indoleamine 2,3-dioxygenase 1 (IDO1). In the mouse model of DSS induced colitis, IDO1 increased levels of pro-inflammatory chemokines and cytokines [99]. We found that the same pathway was up-regulated in SSA/Ps. However, generally IDO is considered as immunosuppressive and has been linked with impaired immune clearance of tumor cells: its activity promotes apoptosis of T-cells, NK cells and induces the differentiation of T regulatory cells (T_regs_) [100, 101]. We hypothesize that IDO1 may play a role in the progression of SSA/Ps to dysplasia and invasive cancer by increasing inflammation and facilitating immune escape. At the pathway level, ‘Inflammatory response’ and ‘Immunological synapse’ were also up-regulated in SSA/Ps as compared to HPs and CR specimens. Other important up-regulated genes and pathways differentiating SSA/Ps from HPs involve cell motility, migration ability, EMT and ECM interaction (Fig. 4, Table 1) that impact cell invasive and metastatic behavior, another hallmark of cancer. Considering pathways differentially co-expressed between SSA/Ps and HPs we found that hub genes were always different between two types of polyps (Additional file 2: Figures S6-S11). For two differentially co-expressed meiosis-related pathways, ‘Meiosis I’ and ‘Meiotic recombination’, the shift in hub genes was from RAD51 (HPs) to MRE11A (SSA/Ps), notably involved in non-homologous recombination and the mismatch repair pathway. The involvement of deficient mismatch repair (dMMR) pathway (that includes MRE11) in CRC is well-documented [102]. Recently, the truncated MRE11 polypeptide was found to be a significant prognostic marker for long-term survival and response to treatment of patients with CRC stage III [103]. Whether SSA/Ps indeed result in the dMMR colon cancer subtype remains to be proven. For ‘Golgi stack’ pathway, the shift from RAB14 toward B3GALT6, essential for the mucopolysaccharides synthesis corresponded to known phenotypic differences between HPs and SSA/Ps. These cases illustrate the ability of our GSNCA tool to confirm existing knowledge, generate new testable hypotheses and raise interesting questions. Based on the up-regulation of the pathways and genes described, we hypothesize that SSA/Ps are prone to neoplastic progression because of an inflammatory and immune escape state, as well as increased cell motility and migration activities.

Using RNA-seq data sets and the new feature selection strategy in combination with popular shrunken centroid classifier (SCC) [38], we developed a gene expression molecular classifier for SSA/Ps that is applicable to microarray data, RNA-seq or qRT-PCR analysis of specimens. The smallest successful signature for all platforms (13 genes) included up-regulated genes previously associated with invasive cell activities (CLDN1, PLA2G16, PTAFR, SPIRE1) and down-regulated checkpoints controlling cell growth (CHFR, NTRK2). In addition, we developed a simple procedure that uses the MAD-normalized signatures to classify new samples as either HP or SSA/P and provides a class probability for the decision, estimated using Cantelli’s inequality. For high throughput platforms where thousands of genes are profiled, it is possible to calculate the Cantelli lower bound for SSA/P and HP probabilities. For other clinical settings that profile a few genes (such as qRT-PCR), accurate classification is also possible but without class assignment probabilities. The proposed molecular classifier for SSA/Ps demonstrated diagnostic value in an independent verification set of specimens and will be further tested to classify specimens profiled with microarray, RNA-seq, or qRT-PCR platforms. The more accurate diagnosis of patients with SSA/Ps will enable future studies that better defines the risk of colon cancer in these patients, determines if subsets of patients have stratified risks for colon cancer and refines the recommendations for follow up care of patients with SSA/Ps [15].

## Conclusions

HPs and SSA/Ps have overlapping histopathological features, yet only SSA/Ps have malignant potential. Our analysis identified that many genes and pathways, up-regulated in SSA/Ps as compared to HPs, CR specimens were involved in inflammatory processes and immune response, suggesting that at the molecular level the presence of inflammation and immunosuppression may constitute the key difference between the two types of polyps. Other genes and pathways, up-regulated in SSA/Ps as compared to HPs, CR specimens included those, related to EMT and ECM interaction, cell motility and migration. Interestingly genes and pathways, differentially expressed and co-expressed between SSA/Ps and HPs, CR specimens constitute known hallmarks of cancer, thus explaining why despite similar histopathological features SSA/Ps have malignant potential.

To objectively differentiate SSA/Ps form HPs we developed a molecular classifier that is platform independent and has low classification error rate for high-throughput data (microarrays, RNA-seq) as well as in small settings (qRT-PCR). We believe our classifier will facilitate further progress with SSA/Ps correct clinical diagnosis.

## Additional files

Additional file 1: **Table S1.** The list of qRT-PCR primers for signature genes. **Table S2.** Normalized expression levels (median and MAD) obtained by qRT-PCR from 45 independent FFPE samples and the classification result obtained using the 13 genes molecular signature with different sample normalizations. **Table S3.** Raw expression levels of 13 genes in the molecular signature obtained by qRT-PCR from 45 independent FFPE samples. **Table S4.** The list of 139 genes that were DE between SSA/Ps and both HP and CR samples. **Table S5.** The list of 172 genes, exclusively DE between SSA/P and HP samples. **Table S6.** The list of 1014 genes, exclusively DE between SSA/P and CR samples. **Table S7.** The list of 105 genes, DE in three comparisons. **Table S8.** Number of common genes between three different platforms. **Table S9.** Class probabilities assigned using empirical approach, normal approximation, shrunken centroid classifier (independent of the summary metric), and the Cantelli’s inequality lower bound when the 18-gene signature from Table 2 is used. **Table S10.** Class probabilities assigned using empirical approach, normal approximation, shrunken centroid classifier (independent of the summary metric), and the Cantelli’s inequality lower bound when the 16-gene signature from Table 2 is used. **Table S11.** Class probabilities assigned using empirical approach, normal approximation, shrunken centroid classifier (independent of the summary metric), and the Cantelli’s inequality lower bound when the 13-gene signature from Table 2 is used. (XLSX 275 kb)

Additional file 2: **Figure S1.** Histograms of the Pearson correlation coefficients between different platforms. **Figure S2.** Barplot of the average raw expression levels of 13 genes obtained by qRT-PCR from 45 FFPE tissue samples. **Figure S3.** Boxplots for the expression levels of 13 genes obtained by qRT-PCR from 45 FFPE tissue samples. **Figure S4.** Histograms of the MAD-normalized log-scale gene expression data in all three platforms. **Figure S5.** Histograms of the summary metric of random signatures of 15 genes in all three platforms. **Figure S6.** MST2 of the ‘Meiosis’ gene set of the C5 collection obtained from MSigDB. **Figure S7.** MST2 of the ‘Regulation of DNA replication’ gene set of the C5 collection obtained from MSigDB. **Figure S8.** MST2 of the ‘Protein targeting to membrane’ gene set of the C5 collection obtained from MSigDB. **Figure S9.** MST2 of the ‘Meiotic recombination’ gene set of the C5 collection obtained from MSigDB. **Figure S10.** MST2 of the ‘Kinase activator activity’ gene set of the C5 collection obtained from MSigDB. **Figure S11.** MST2 of the ‘Hormone activity’ gene set of the C5 collection obtained from MSigDB. **Figure S12.** Scatter plot of the first and second principal components for normalized expression levels. (PDF 151 kb)

Additional file 3: Text S1. R code with instructions on how to classify new qRT-PCR samples as HP or SSA/P. (R 1 kb)

## Abbreviations

AUC: Area under ROC curve
CIMP: CpG island methylator phenotype
CL: Control left
CLB: Cantelli lower bound
CR: Control right
CRC: Colorectal cancer
DE: Differentially expressed
ECM: Extracellular matrix
EMT: Epithelial-mesenchymal transition
FC: Fold change
FFPE: Formalin-fixed paraffin-embedded
FPKM: Fragments per kilobase per millions
GEO: Gene expression omnibus
GO: Gene ontology
GSNCA: Gene sets net correlations analysis
HP: Hyperplastic polyps
MSI: Microsatellite instability
MSigDB: Molecular signature database
MVHP: Microvesicular hyperplastic polyps
qRT-PCR: quantitative real-time polymerase chain reaction
SCC: Shrunken centroid classifier
SM: Summary metric
SSA: Sessile serrated adenoma
SSA/Ps: Sessile serrated adenoma/polyps
