# Supplementary material for: Platform-independent gene expression signature differentiates sessile serrated adenomas/polyps and hyperplastic polyps of the colon
Source: BMC Med Genomics. 2017 Dec 28;10:81. doi: 10.1186/s12920-017-0317-7 (PMC5745747; doi:10.1186/s12920-017-0317-7)
Supplement: Supplementary file 2 — Figure S1. Histograms of the Pearson correlation coefficients between different platforms. Figure S2. Barplot of the average raw expression levels of 13 genes obtained by qRT-PCR from 45 FFPE tissue samples. Figure S3. Boxplots for the expression levels of 13 genes obtained by qRT-PCR from 45 FFPE tissue samples. Figure S4. Histograms of the MAD-normalized log-scale gene expression data in all three platforms. Figure S5. Histograms of the summary metric of random signatures of 15 genes in all three platforms. Figure S6. MST2 of the ‘Meiosis’ gene set of the C5 collection obtained from MSigDB. Figure S7. MST2 of the ‘Regulation of DNA replication’ gene set of the C5 collection obtained from MSigDB. Figure S8. MST2 of the ‘Protein targeting to membrane’ gene set of the C5 collection obtained from MSigDB. Figure S9. MST2 of the ‘Meiotic recombination’ gene set of the C5 collection obtained from MSigDB. Figure S10. MST2 of the ‘Kinase activator activity’ gene set of the C5 collection obtained from MSigDB. Figure S11. MST2 of the ‘Hormone activity’ gene set of the C5 collection obtained from MSigDB. Figure S12. Scatter plot of the first and second principal components for normalized expression levels. (PDF 151 kb) [file 12920_2017_317_MOESM2_ESM.pdf]

Platform-independent Gene Expression Signature Differentiates Sessile Serrated Adenomas/Polyps and Hyperplastic Polyps of the Colon (Additional file 2 - Supplementary Figures)

Yasir Rahmatallah [1], Magomed Khaidakov [2,3]
Keith Lai [5], Hannah Goyne [4], Laura Lamps [4]
Curt Hagedorn [2,3] and Galina Glazko [1]

[1] Department of Biomedical Informatics,
University of Arkansas for Medical Sciences,
Little Rock, AR 72205.
[2] The Central Arkansas Veterans Healthcare System,
Little Rock, AR 72205.
[3] Department of Medicine, Division of Gastroenterology and Hepatology,
University of Arkansas for Medical Sciences,
Little Rock, AR 72205.
[4] Department of Pathology,
University of Arkansas for Medical Sciences, Little Rock, AR 72205.
[5] Department of Anatomic Pathology,
Cleveland Clinic, Cleveland, OH 44195.

December 20, 2017

List of Figures

S1 Histograms of Pearson correlation coefficients between different platforms. 4
S2 Barplot of the average raw expression levels of 13 genes obtained by qRT-PCR from 45 FFPE tissue samples. 5
S3 Boxplots for the expression levels of 13 genes obtained by qRT-PCR from 45 FFPE tissue samples. 6
S4 Histograms of the MAD-normalized log-scale gene expression data in all three platforms. 7
S5 Histograms of the summary metric of random signatures of 15 genes in all three platforms. 8
S6 MST2 of the MEIOSIS gene set of the C5 collection obtained from MSigDB. 9
S7 MST2 of the REGULATION OF DNA REPLICATION gene set of the C5 collection obtained from MSigDB. 10
S8 MST2 of the PROTEIN TARGETING TO MEMBRANE gene set of the C5 collection obtained from MSigDB. 11
S9 MST2 of the MEIOTIC RECOMBINATION gene set of the C5 collection obtained from MSigDB. 12
S10 MST2 of the KINASE ACTIVATOR ACTIVITY gene set of the C5 collection obtained from MSigDB. 13
S11 MST2 of the HORMONE ACTIVITY gene set of the C5 collection obtained from MSigDB. 14
S12 Scatter plot of the first and second principal components for normalized expression levels. 15

Introduction

This section provides details regarding the supplementary Figures and how they were generated.

Figure S1 shows histograms of Pearson correlation coefficients between two platforms obtained in 10000 iterations. Only 117 genes expressed in all three platforms (RNA-seq, Illumina, and Affymetrix) and found to be differentially expressed between SSA/Ps and both HPs and CRs are considered. Panel (A) shows the histogram of Pearson's correlation coefficient between the RNA-seq and the Illumina platforms when phenotypic labels are preserved. Panel (B) shows the histogram of Pearson's correlation coefficient between the RNA-seq and the Illumina platforms when phenotypic labels are randomly resampled. Panel (C) shows the histogram of Pearson's correlation coefficient between the RNA-seq and the Affymetrix platforms when phenotypic labels are preserved. Panel (D) shows the histogram of Pearson's correlation coefficient between the RNA-seq and the Affymetrix platforms when phenotypic labels are randomly resampled.

Figure S2 shows barplot of the average raw expression levels of 13 genes obtained by quantitative real-time polymerase chain reaction (qRT-PCR) from 45 formalin-fixed paraffin-embedded (FFPE) tissue samples. For each gene samples are grouped according to their phenotype (HP or SSA/P). Error bars extend to ± one standard deviation. Raw expression levels are relative to the housekeeping gene, hence higher levels here refer to lower values.

Figure S3 shows boxplots for the expression levels of 13 genes obtained by quantitative real-time polymerase chain reaction (qRT-PCR) from 45 formalin-fixed paraffin-embedded (FFPE) tissue samples. Panel (A) shows raw expression levels centered around zero. Panel (B) shows normalized expression levels by first subtracting sample medians and then by subtracting gene-wise medians from each individual gene.

Figure S4 shows that histograms of the MAD-normalized log-scale gene expression data in all three platforms approximately follow a Laplace-like distribution centered around zero. Panel (A) shows the histogram of the RNA-seq dataset (17243 genes and 31 samples). Panel (B) shows the histogram of the Illumina dataset (17123 genes and 12 samples). Panel (C) shows the histogram for the Affymetrix dataset (19090 genes and 17 samples).

Figure S5 shows histograms of the summary metric (SM) obtained by summing the MAD-normalized expressions of a random signature of 15 genes in all three platforms. Six HP and six SSA/P samples were randomly selected from each platform in each iteration and a total of 10000 iterations were used to generate the histogram of SM. The SM approximately follows a normal-like distribution that is centered around zero and has a higher kurtosis than the standard normal distribution. Panel (A) shows the RNA-seq data set. Panel (B) shows the Illumina data set. Panel (C) shows the Affymetrix data set.

Figure S6 shows the union of the first and second minimum spanning trees (MST2) for the MEIOSIS gene set of the C5 collection obtained from the molecular signature database (MSigDB). This gene set is detected by gene sets net correlations analysis (GSNCA) at $P < 0.05$ in both comparisons: HP versus SSA/P and CR versus SSA/P.

Figure S7 shows the union of the first and second minimum spanning trees (MST2) for the REGULATION OF DNA REPLICATION gene set of the C5 collection obtained from the molecular

signature database (MSigDB). This gene set is detected by gene sets net correlations analysis (GSNCA) at $P < 0.05$ in both comparisons: HP versus SSA/P and CR versus SSA/P.

Figure S8 shows the union of the first and second minimum spanning trees (MST2) for the PROTEIN TARGETING TO MEMBRANE gene set of the C5 collection obtained from the molecular signature database (MSigDB). This gene set is detected by gene sets net correlations analysis (GSNCA) at $P < 0.05$ in both comparisons: HP versus SSA/P and CR versus SSA/P.

Figure S9 shows the union of the first and second minimum spanning trees (MST2) for the MEIOTIC RECOMBINATION gene set of the C5 collection obtained from the molecular signature database (MSigDB). This gene set is detected by gene sets net correlations analysis (GSNCA) at $P < 0.05$ in both comparisons: HP versus SSA/P and CR versus SSA/P.

Figure S10 shows the union of the first and second minimum spanning trees (MST2) for the KINASE ACTIVATOR ACTIVITY gene set of the C5 collection obtained from the molecular signature database (MSigDB). This gene set is detected by gene sets net correlations analysis (GSNCA) at $P < 0.05$ in both comparisons: HP versus SSA/P and CR versus SSA/P.

Figure S11 shows the union of the first and second minimum spanning trees (MST2) for the HORMONE ACTIVITY gene set of the C5 collection obtained from the molecular signature database (MSigDB). This gene set is detected by gene sets net correlations analysis (GSNCA) at $P < 0.05$ in both comparisons: HP versus SSA/P and CR versus SSA/P.

Figure S12 shows the scatter plot of the first and second principle components for normalized expression levels. Raw expression levels were first normalized by subtracting sample medians and then by subtracting gene-wise medians from each individual gene.

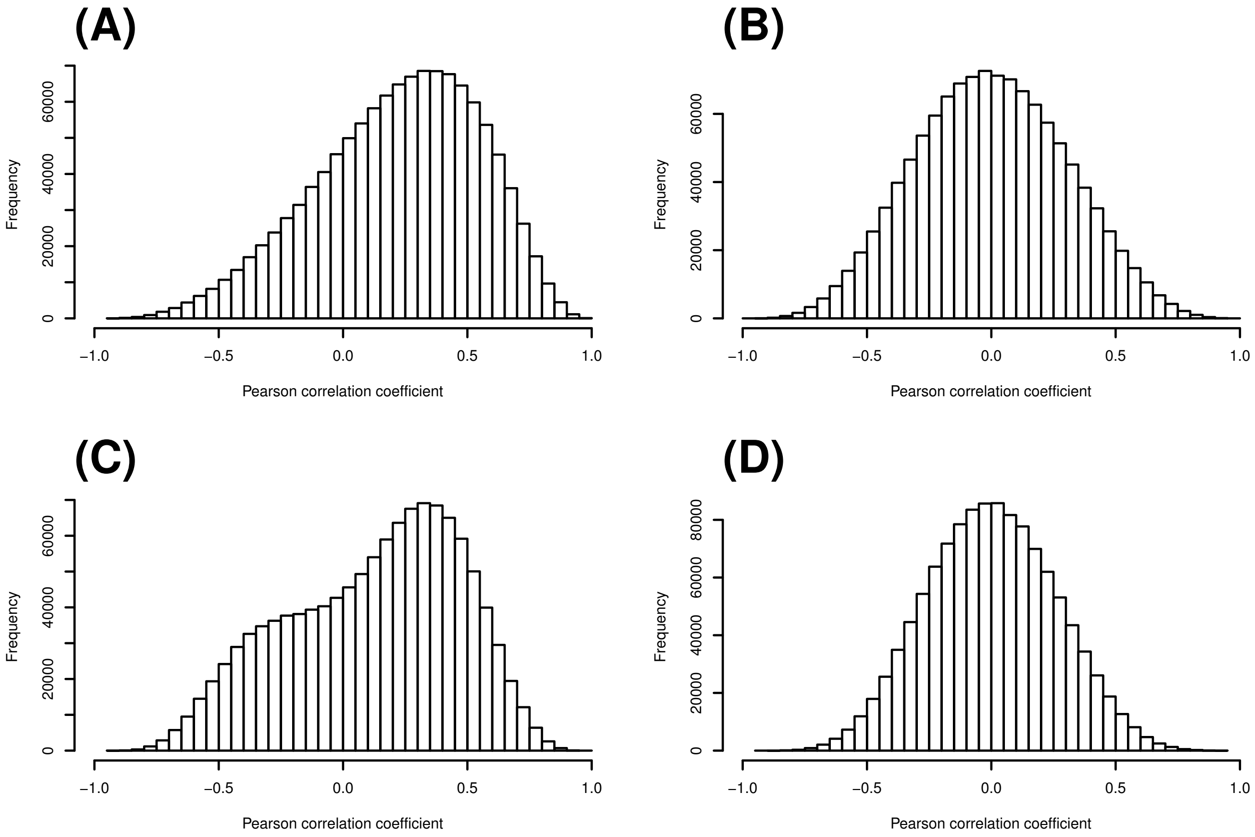

Figure S1: Histograms of Pearson correlation coefficients between different platforms.

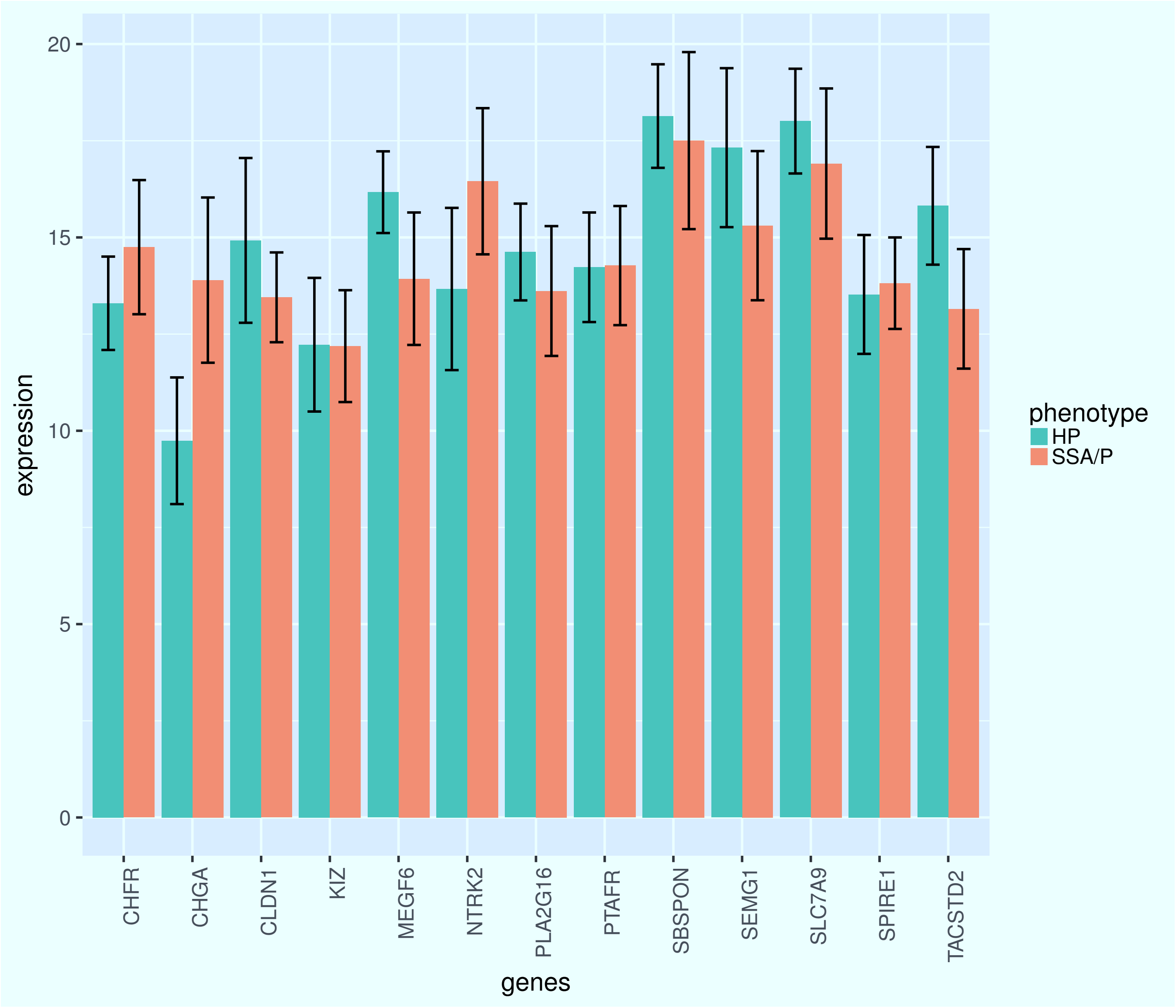

Figure S2: Barplot of the average raw expression levels of 13 genes obtained by qRT-PCR from 45 FFPE tissue samples.

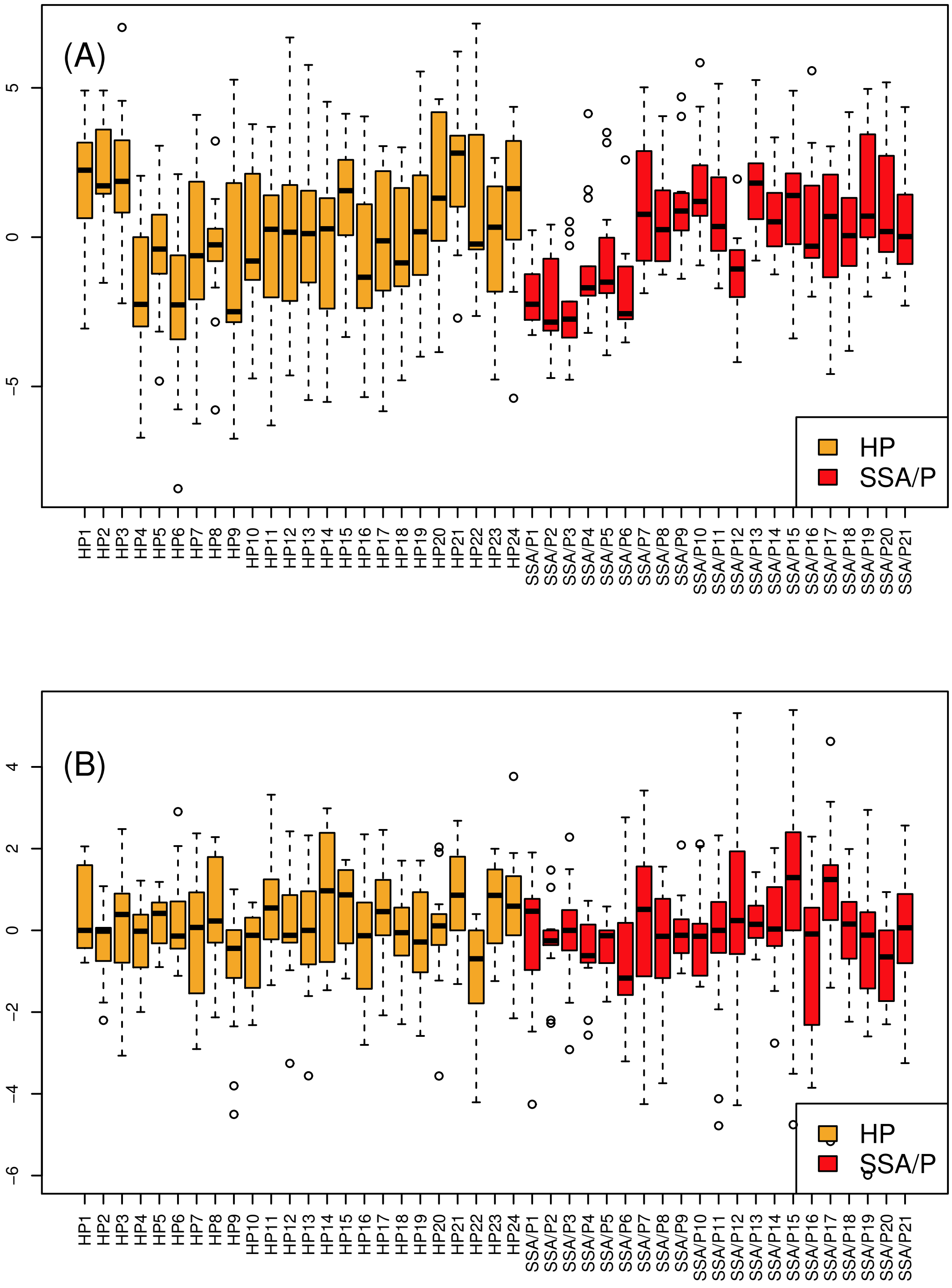

Figure S3: Boxplots for the expression levels of 13 genes obtained by qRT-PCR from 45 FFPE tissue samples.

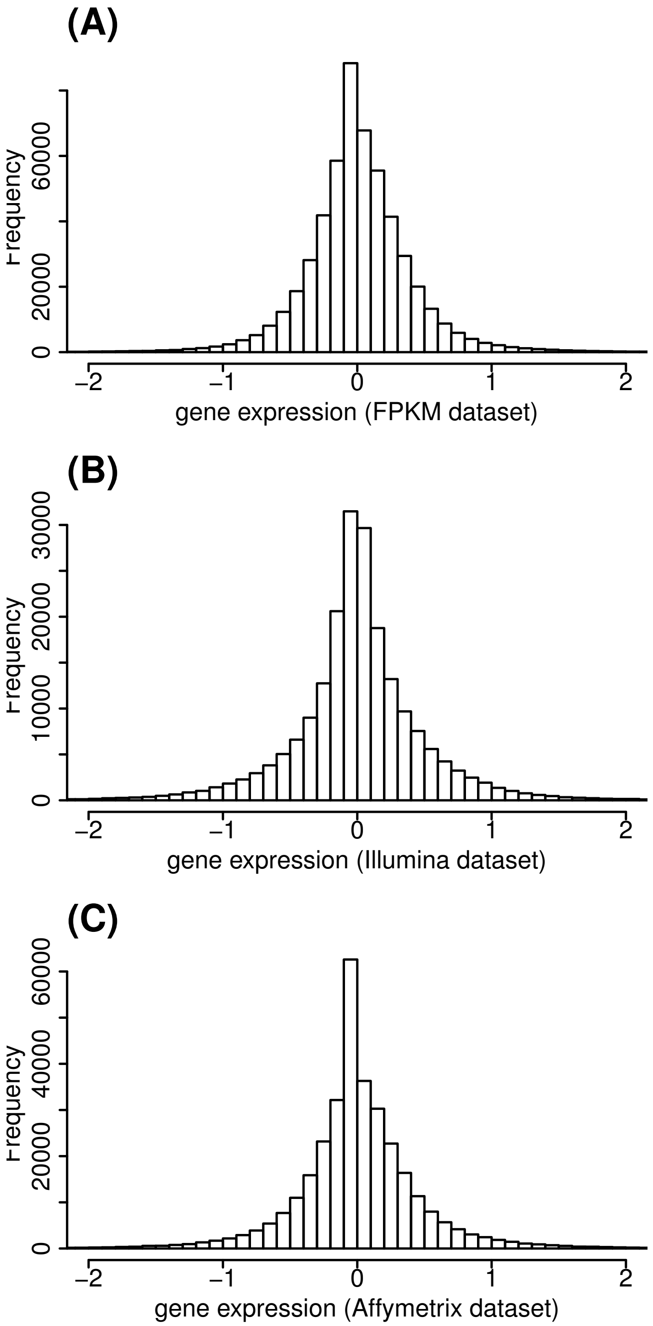

Figure S4: Histograms of the MAD-normalized log-scale gene expression data in all three platforms.

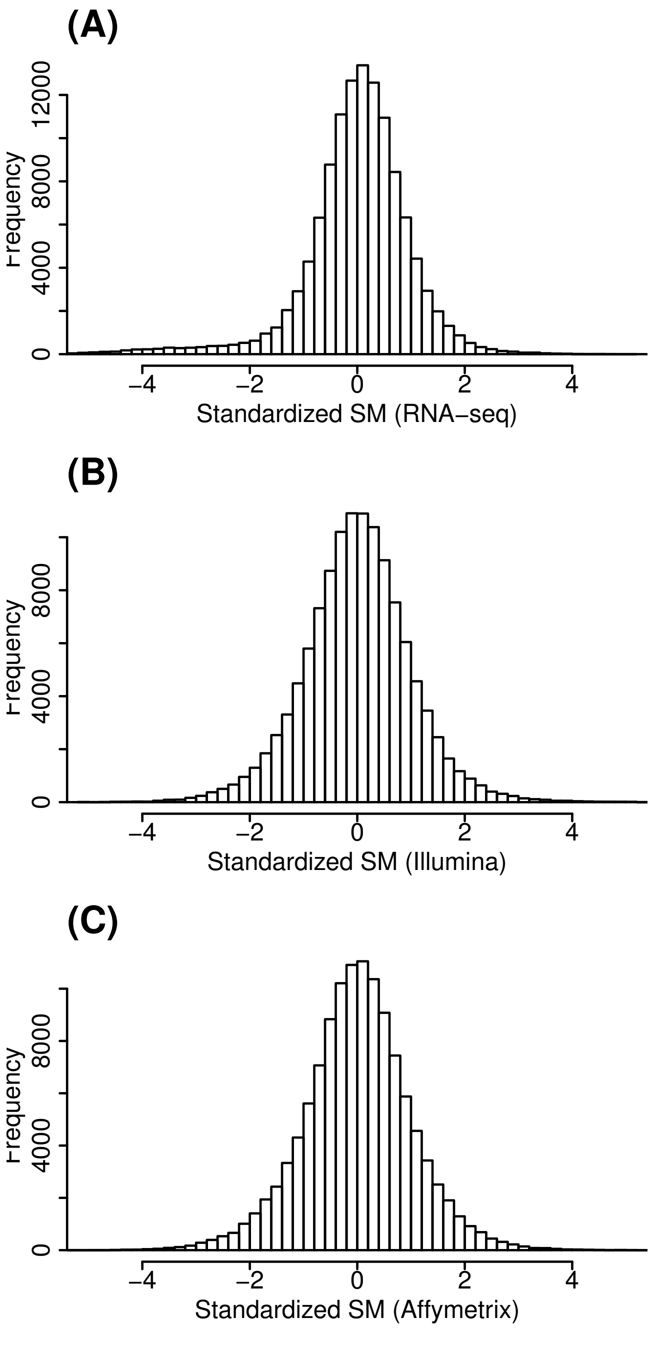

Figure S5: Histograms of the summary metric of random signatures of 15 genes in all three platforms.

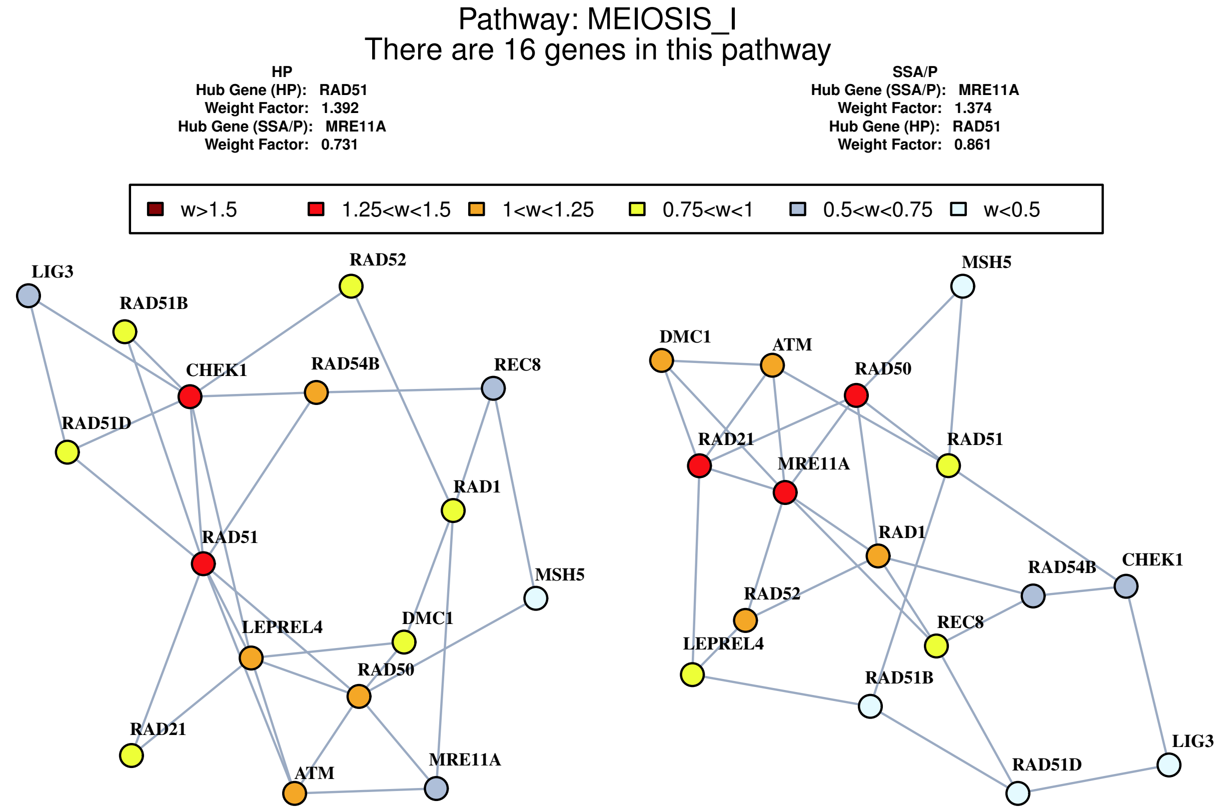

Figure S6: MST2 of the MEIOSIS gene set of the C5 collection obtained from MSigDB.

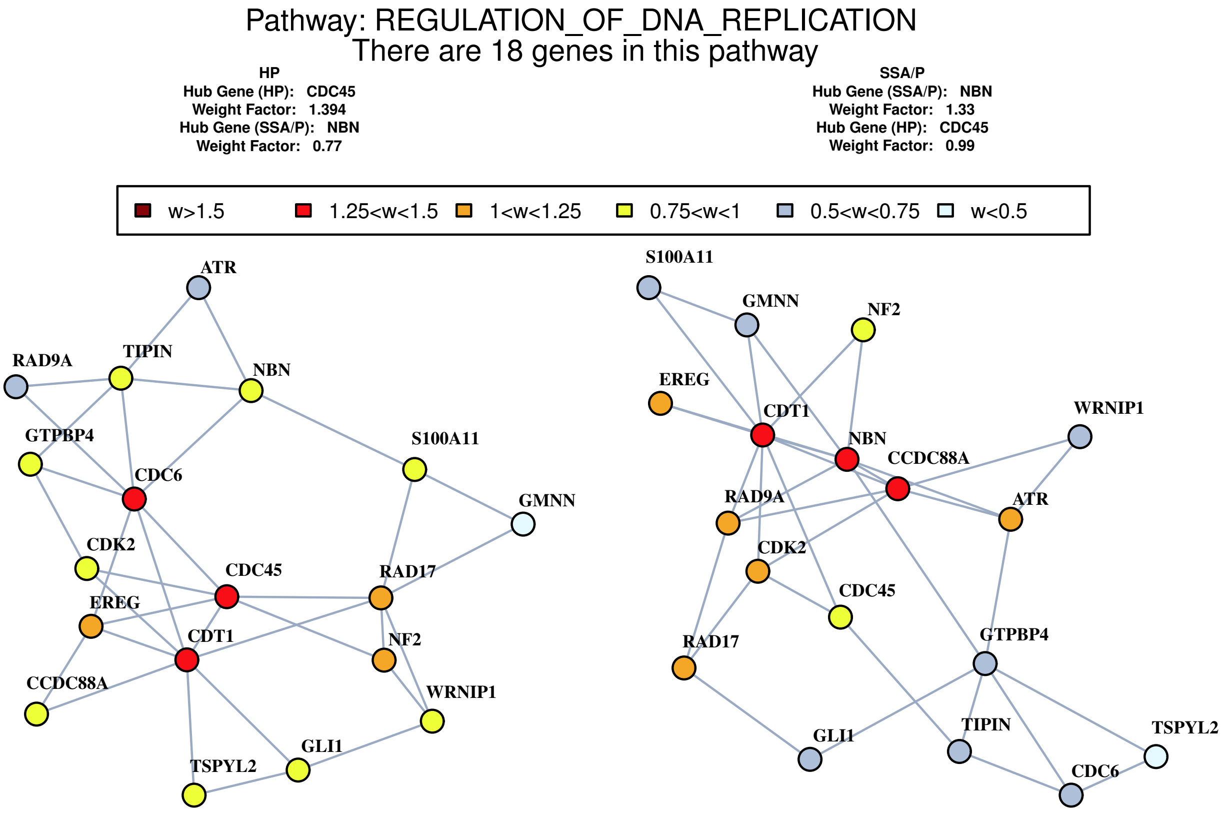

Figure S7: MST2 of the REGULATION OF DNA REPLICATION gene set of the C5 collection obtained from MSigDB.

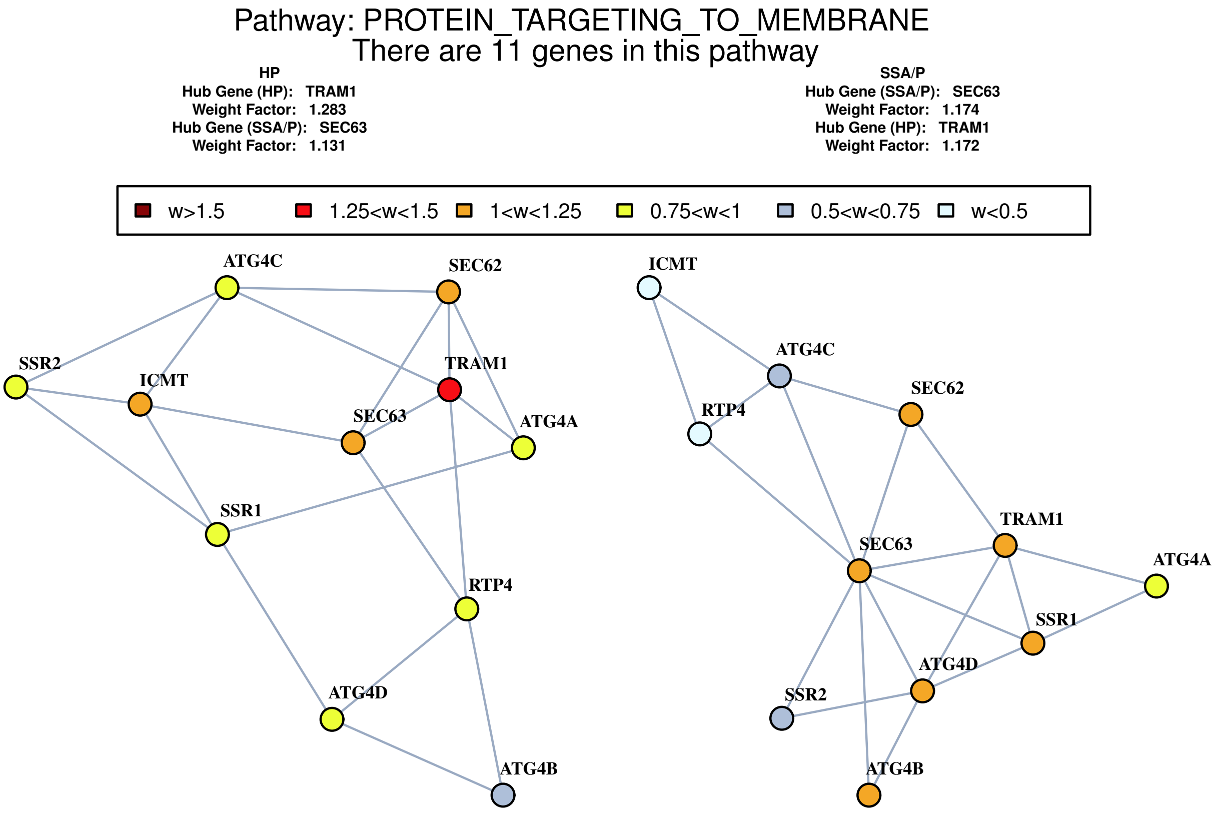

Figure S8: MST2 of the PROTEIN TARGETING TO MEMBRANE gene set of the C5 collection obtained from MSigDB.

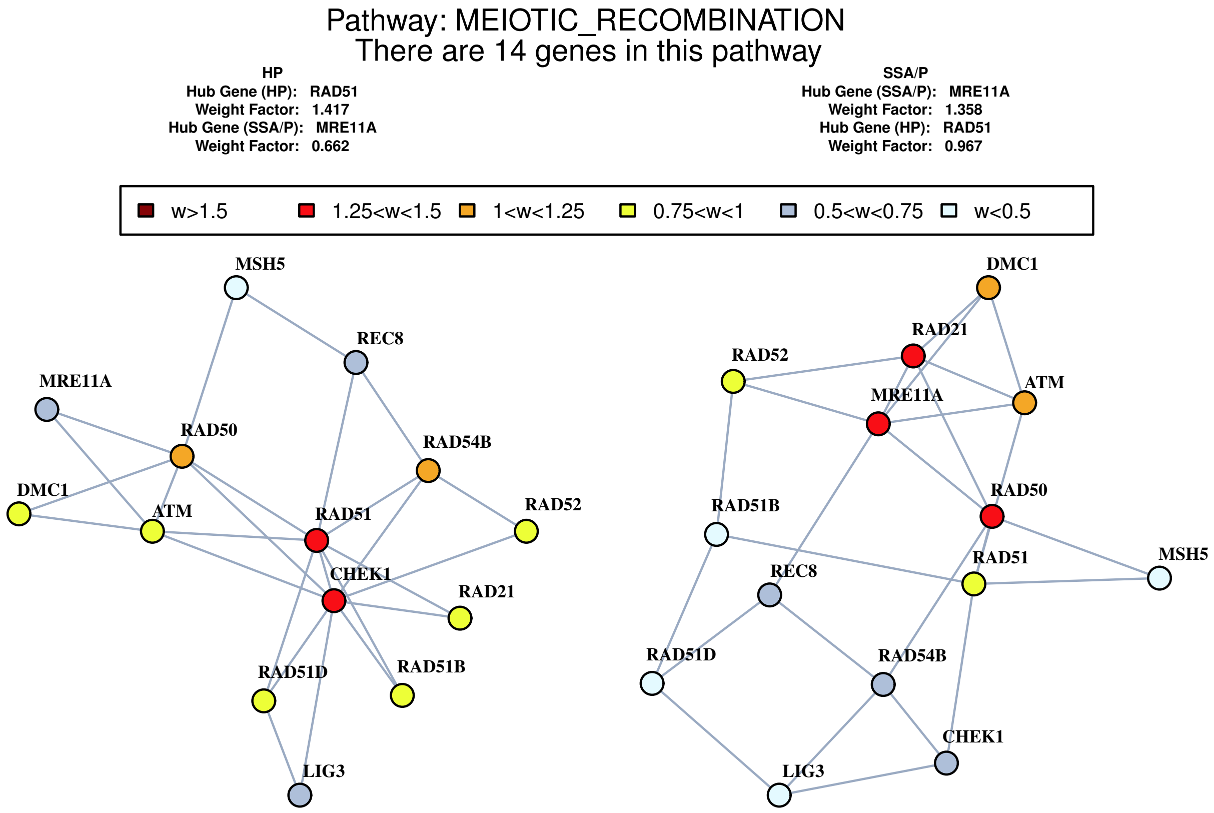

Figure S9: MST2 of the MEIOTIC RECOMBINATION gene set of the C5 collection obtained from MSigDB.

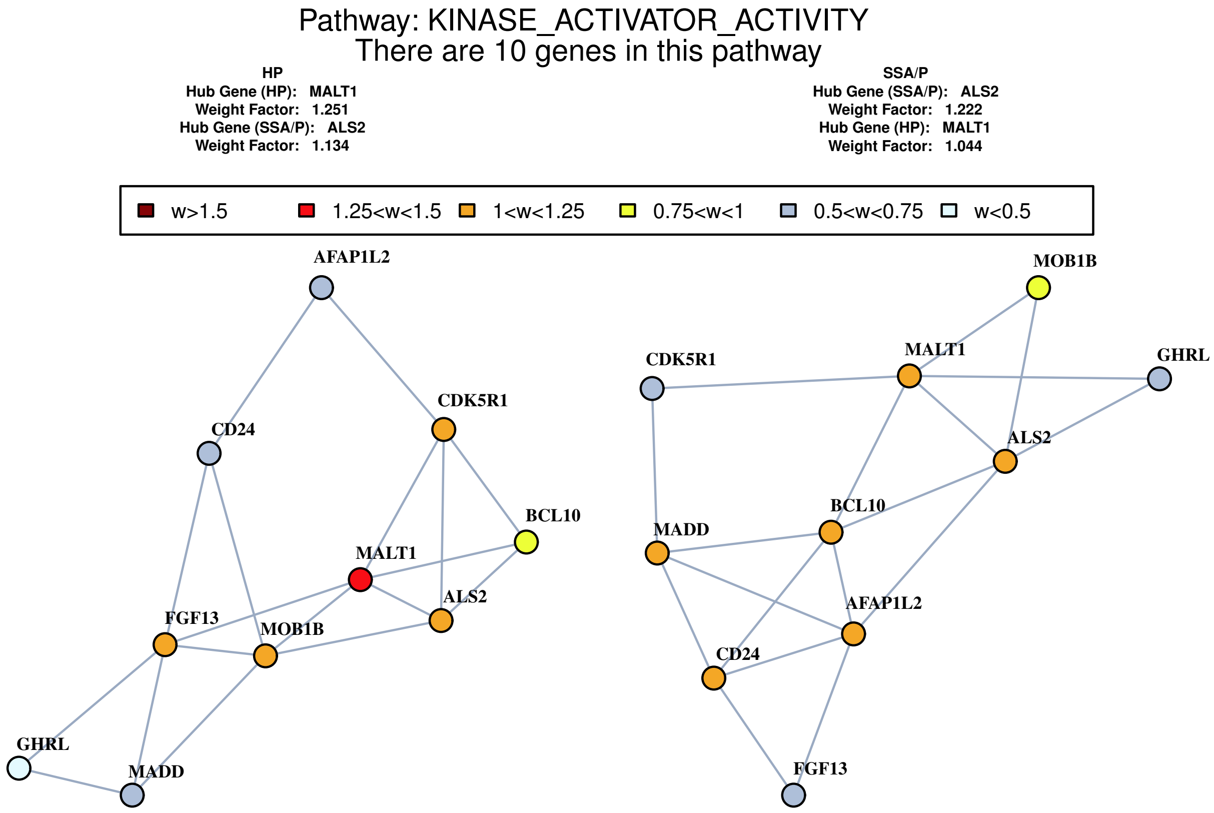

Figure S10: MST2 of the KINASE ACTIVATOR ACTIVITY gene set of the C5 collection obtained from MSigDB.

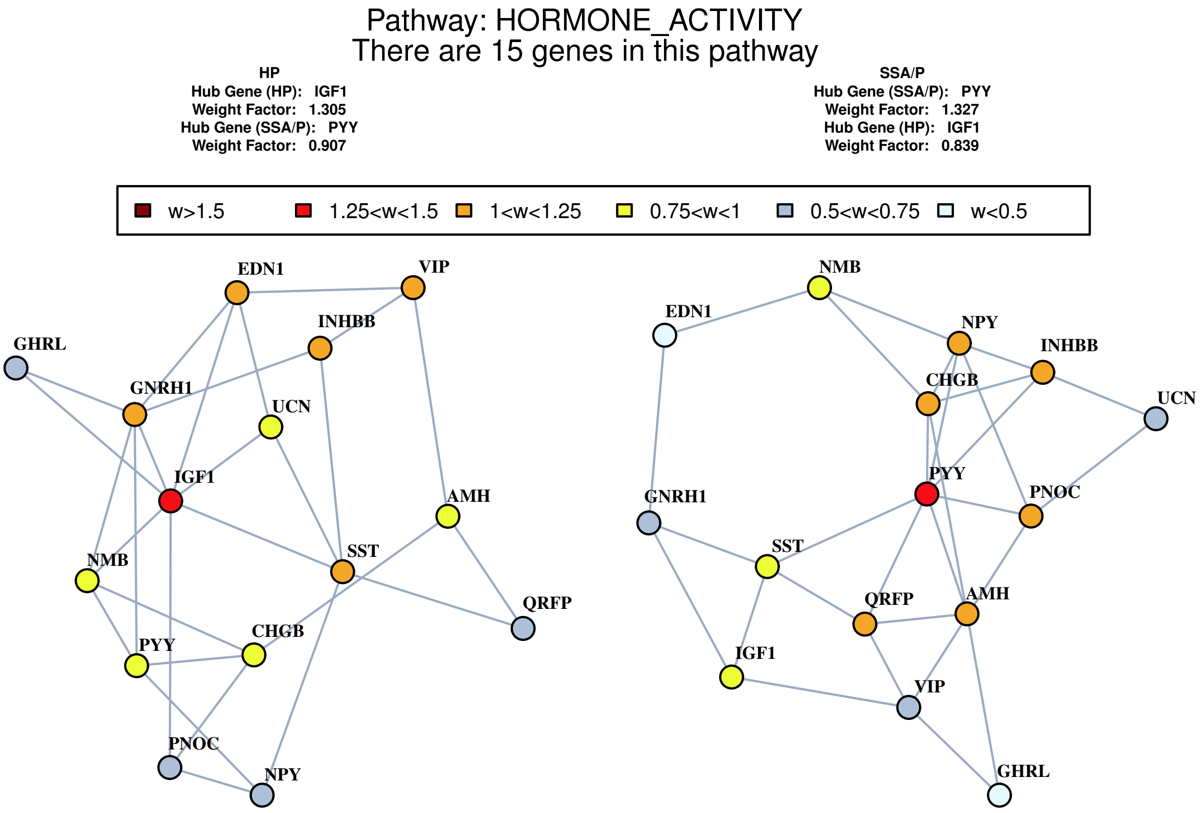

Figure S11: MST2 of the HORMONE ACTIVITY gene set of the C5 collection obtained from MSigDB.

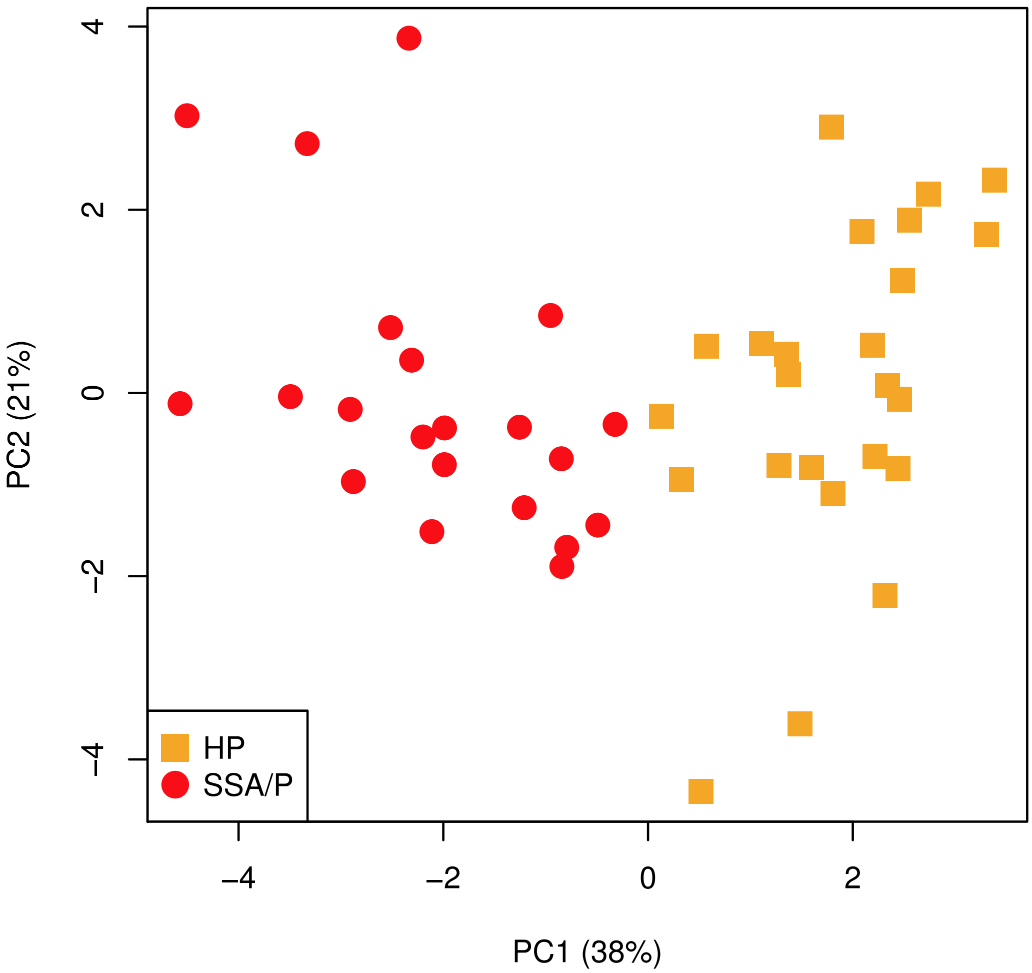

Figure S12: Scatter plot of the first and second principal components for normalized expression levels.
